# Structures of pMV158 replication initiator RepB with and without DNA reveal a flexible dual-function protein

**DOI:** 10.1093/nar/gkac1271

**Published:** 2023-01-23

**Authors:** Cristina Machón, José A Ruiz-Masó, Juliana Amodio, D Roeland Boer, Lorena Bordanaba-Ruiseco, Katarzyna Bury, Igor Konieczny, Gloria del Solar, Miquel Coll

**Affiliations:** Institute for Research in Biomedicine (IRB Barcelona), The Barcelona Institute of Science and Technology (BIST), Baldiri Reixac 10-12, 08028 Barcelona, Spain; Institut de Biologia Molecular de Barcelona (IBMB-CSIC), Baldiri Reixac 10-12, 08028 Barcelona, Spain; Centro de Investigaciones Biológicas Margarita Salas (CIB-CSIC), Ramiro de Maeztu 9, 28040 Madrid, Spain; Institute for Research in Biomedicine (IRB Barcelona), The Barcelona Institute of Science and Technology (BIST), Baldiri Reixac 10-12, 08028 Barcelona, Spain; Institut de Biologia Molecular de Barcelona (IBMB-CSIC), Baldiri Reixac 10-12, 08028 Barcelona, Spain; Institute for Research in Biomedicine (IRB Barcelona), The Barcelona Institute of Science and Technology (BIST), Baldiri Reixac 10-12, 08028 Barcelona, Spain; Institut de Biologia Molecular de Barcelona (IBMB-CSIC), Baldiri Reixac 10-12, 08028 Barcelona, Spain; Centro de Investigaciones Biológicas Margarita Salas (CIB-CSIC), Ramiro de Maeztu 9, 28040 Madrid, Spain; Intercollegiate Faculty of Biotechnology of University of Gdansk and Medical University of Gdansk, University of Gdansk, Abrahama 58, 80-307 Gdansk, Poland; Intercollegiate Faculty of Biotechnology of University of Gdansk and Medical University of Gdansk, University of Gdansk, Abrahama 58, 80-307 Gdansk, Poland; Centro de Investigaciones Biológicas Margarita Salas (CIB-CSIC), Ramiro de Maeztu 9, 28040 Madrid, Spain; Institute for Research in Biomedicine (IRB Barcelona), The Barcelona Institute of Science and Technology (BIST), Baldiri Reixac 10-12, 08028 Barcelona, Spain; Institut de Biologia Molecular de Barcelona (IBMB-CSIC), Baldiri Reixac 10-12, 08028 Barcelona, Spain

## Abstract

DNA replication is essential to all living organisms as it ensures the fidelity of genetic material for the next generation of dividing cells. One of the simplest replication initiation mechanisms is the rolling circle replication. In the streptococcal plasmid pMV158, which confers antibiotic resistance to tetracycline, replication initiation is catalysed by RepB protein. The RepB N-terminal domain or origin binding domain binds to the recognition sequence (*bind* locus) of the double-strand origin of replication and cleaves one DNA strand at a specific site within the *nic* locus. Using biochemical and crystallographic analyses, here we show how the origin binding domain recognises and binds to the *bind* locus using structural elements removed from the active site, namely the recognition α helix, and a β-strand that organises upon binding. A new hexameric structure of full-length RepB that highlights the great flexibility of this protein is presented, which could account for its ability to perform different tasks, namely bind to two distinct loci and cleave one strand of DNA at the plasmid origin.

## Graphical Abstract

**Graphical Abstract:**
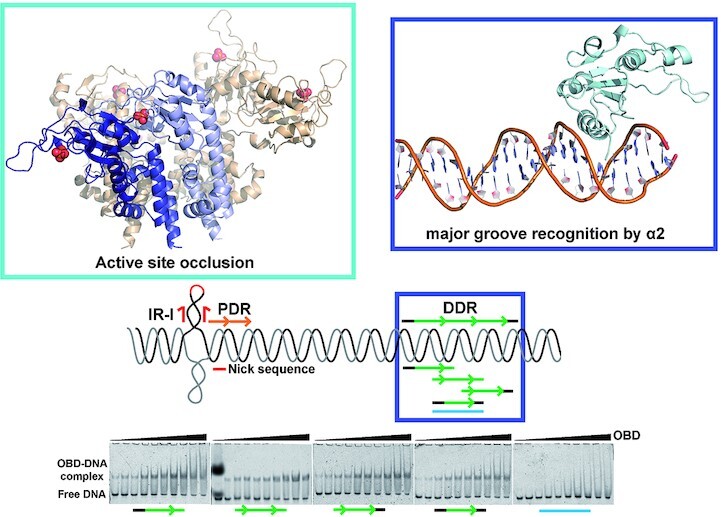
Here, we report a new 3D structure of plasmid pMV158 replication initiator RepB6, showing the great versatility of this protein, as well as the accessible and occluded catalytic active sites within it. We have also characterised biochemically the binding of the OBD to the DDR and solved the structure of the OBD–DDR complex.

## INTRODUCTION

One of the critical steps in DNA replication is the supply of a free OH group to DNA polymerases, which is necessary for starting the synthesis of a new strand of DNA. Initiation of rolling circle replication (RCR) requires the replication initiation protein Rep to interact with specific DNA sequences within the plasmid called the double-strand origin (*dso*). The Rep protein binds to the *bind* locus and cleaves a specific phosphodiester bond within the nick sequence. As a result of this reaction, the Rep protein becomes covalently bound to the newly generated 5′-P end, leaving a 3′-OH free, available for the DNA polymerase to initiate leading-strand replication. After a complete round of replication, a new cleavage episode, presumably catalysed by the Rep protein covalently attached to the DNA, takes place at the reconstituted nick site. The following strand-transfer reaction results in a dsDNA plasmid molecule and a ssDNA intermediate that corresponds to the recircularised cleaved parental strand. The formation of ssDNA intermediates is a hallmark of plasmid RCR (1).

RCR has been largely characterised in plasmids from Gram-positive bacteria, such as the streptococcal promiscuous plasmid pMV158, which contains a tetracycline antibiotic resistance gene. RepB, the replication initiation protein of pMV158, is a member of the HUH endonuclease superfamily (2), which includes proteins involved in the replication of plasmids, bacteriophages and viruses, as well as proteins with roles in DNA transposition and in conjugative mobilisation of plasmids (3). These proteins are characterised by the presence of two conserved protein motifs: the HUH motif, composed of two His residues separated by a bulky hydrophobic amino acid, which is responsible for the coordination of a divalent metal cation, and the catalytic motif, containing one or two Tyr residues separated by several amino acids (3). RepB from plasmid pMV158 is one of the members of the RCR group of HUH endonucleases whose crystal structure has been determined (4). RepB is purified as a homohexamer, an exclusive feature that distinguishes it from other Rep proteins from plasmids and bacteriophages, which have been purified as monomers or dimers (5,6).

Each monomer of RepB comprises two domains connected by a short hinge region (4). The C-terminal or oligomerisation domain (OD) is a helical domain responsible for the hexameric state of the protein, while the N-terminal or origin binding domain (OBD) carries the catalytic active site and is in charge of recognising, cleaving and joining *dso* DNA (7). Both domains retain their specific abilities when purified separately (4). While the ODs form a toroidal ring with near six-fold symmetry in the two crystal structures previously available (4), the OBDs arrange either as a dimer of trimers (in the trigonal crystal form) or as a trimer of dimers (in the tetragonal crystal form). Superposition of these two structures through the OD shows differences of up to 55° in the relative orientation of the OBDs. For both structures, the crystals were formed by incubating RepB with a 34-bp DNA fragment comprising the entire pMV158 *bind* region. Although the presence of DNA was confirmed by fluorescence microscopy, the electron density corresponding to DNA near the positively charged surface of the OBD was too weak to be determined (4).

The *bind* locus of the pMV158 *dso* consists of three 11-bp tandem direct repeats, named distal direct repeats (DDRs) because they are located 84 bp downstream of the nick site, which is situated on the loop of a hairpin formed by the inverted repeat IR-I (8). This hairpin and also two 7-bp tandem direct repeats, termed proximal direct repeats (PDRs) due to their immediacy to the nick site, are part of the *nic* locus of the *dso*. RepB of pMV158 binds primarily to the DDRs with more affinity and stability than to the PDRs, and high-resolution dimethyl sulfate (DMS) footprinting shows that the protein interacts through the major groove of the DNA with the same Gs of each DDR from the *bind* locus, protecting them from methylation (9).

Despite all these experiments, there is a lack of information about the requirements for the specific DDR recognition by the OBD, both from the DNA and protein perspectives. Here we present a thorough study of the interaction of RepB OBD with different DNA fragments from the *bind* locus of the pMV158 *dso*, including the crystal structure of the OBD complexed with a 23-bp dsDNA comprising two DDRs of the *bind* locus (9). This structure reveals the main residues involved in the recognition of the plasmid *dso*, as well as the conformational changes in the N-terminal domain of RepB, notably the extension of a β-sheet that accompanies the binding of the protein to its target DNA. We also reveal changes to DNA binding that result from the mutation of crucial amino acids in RepB. Finally, we present a new RepB hexameric structure obtained in the absence of DNA, which shares the same 6-fold symmetry within the ODs, but displays a completely different organisation of the OBDs, due to an enlarged α-helix in the OD in some of the protomers. In addition to reflecting the remarkable (much higher than predicted) interdomain flexibility of RepB, the new structure might explain the capacity of this protein to bind simultaneously to two distant loci and perform its catalytic activity.

## MATERIALS AND METHODS

### Electrophoresis mobility shift assays (EMSAs) and cooperativity experiments

EMSAs to measure the binding affinity of OBD for each of the direct repeat sequences of the *bind* locus (Supplementary Table S1) were performed in buffer B (20 mM Tris–HCl pH 8.0, 5 mM DTT, 300 mM NaCl) containing increasing concentrations of purified OBD, ranging from 0.5 to 50 μM, and 20 nM of the corresponding 5′-Cy5-labelled dsDNA oligonucleotide (oligo). Binding mixtures were incubated 20 min at 25°C, and free and bound DNA was separated by electrophoresis on native 6% polyacrylamide (PAA) gels (30:1 acrylamide:bis-acrylamide; BIO-RAD, 1610146) and quantified using a FLA-3000 (FUJIFILM) imaging system and QuantityOne software (BIO-RAD). For each of the OBD–dsDNA oligo binding analysed, the gels of three independent experiments were quantified. The OBD protein concentration required for half-maximal binding under the described experimental conditions (*K*_d_) was obtained with the Sigmaplot 12.5 software (Systat Software Inc.), from data point fitting to the equation $y\ = \ {B}_{max}x/( {{K}_d + x} )$; where *y* is the fraction of complexed dsDNA, *B_max_* is the maximum fraction of bound DNA, and *x* is the concentration of the OBD. The dsDNA binding activity of the OBD preparation was determined by EMSA at stoichiometric binding conditions, using 1 and 2 μM of purified OBD and 5 μM of DDRL dsDNA. OBD protein preparation was ∼93% active.

The dissociation rate of OBD–DNA complexes was analysed by equilibrating the OBD and the 5′-Cy5-labelled DDRC fragment (Supplementary Table S1) under conditions in which the fraction of complexed DNA was ∼0.5, and then adding (at *t* = 0) a 1000-fold molar excess of the unlabelled DDRC DNA. Samples were taken at intervals and applied to a running native 6% PAA gel. As a control to verify that competition conditions were fulfilled, the OBD was added to a mixture of unlabelled and labelled (molar ratio 1000:1) DDRC DNA and incubated in the same conditions. Less than 5% of the DNA became complexed to the protein, and this value was taken as background for the OBD–DDRC dissociation experiments.

To evaluate the potential cooperative binding of the monomeric OBD to the entire *bind* locus, we performed an EMSA, maintaining a fixed concentration (20 nM) of the fluorescent 3DDR DNA (Supplementary Table S1) and increasing the OBD concentration from 0.1 to 1.3 μM. The analysis and quantification of the gels allowed us to obtain the fractions of DNA with 0, 1, 2 and all 3 sites occupied by the OBD, corresponding to free DNA, and the C1, C2 and C3 complexes, respectively. In a system composed of three interacting DNA binding sites and a single protein ligand that consider only pairwise cooperative interactions (10), the binding equations for Θ_i_, which is the fraction of DNA molecules with exactly *i* ligands bound, are:

(2a) $$\begin{equation*}{{\rm{\theta }}}_0 = \frac{1}{Z}\ \end{equation*}$$

(2b) $$\begin{equation*}{{\rm{\theta }}}_1 = \frac{{\left( {{k}_1 + {k}_2 + {k}_3} \right)L}}{Z}\ = \frac{{{K}_1L}}{Z}\ \end{equation*}$$

(2c) $$\begin{equation*}{{\rm{\theta }}}_2 = \frac{{\left( {{k}_1{k}_2{k}_{12} + {k}_1{k}_3{k}_{13} + {k}_2{k}_3{k}_{23}} \right){L}^2}}{Z}\ = \frac{{{K}_2{L}^2}}{Z}\ \end{equation*}$$

(2d) $$\begin{equation*}{{\rm{\theta }}}_3 = \frac{{\left( {{k}_1{k}_2{k}_3({k}_{12} + {k}_{13} + {k}_{23}} \right)){L}^3}}{Z}\ = \frac{{{K}_3{L}^3}}{Z}\ \end{equation*}$$

where $L$ is the concentration of free protein ligand; $Z$ is the binding polynomial equal to 1 + *K*_1_*L* + *K*_2_*L*^2^ + *K*_3_*L*^3^; *k*_1_, *k*_2_ and *k*_3_ are the microscopic equilibrium association constants for intrinsic binding to sites 1, 2 and 3; and *k*_12_, *k*_13_ and *k*_23_ are the constants describing cooperative interactions when the corresponding sites are liganded. Microscopic equilibrium constants can be replaced by three macroscopic constants defined by *K*_1_ = ${k}_1 + {k}_2 + {k}_3$, *K*_2_ = ${k}_1{k}_2{k}_{12} + {k}_1{k}_3{k}_{13} + {k}_2{k}_3{k}_{23}$ and *K*_3_ = ${k}_1{k}_2{k}_3( {{k}_{12} + {k}_{13} + {k}_{23}} )$. Only these macroscopic equilibrium constants can be determined from a single mobility-shift experiment, and the cooperative interactions are inferred by comparing them. Considering a three-site system, the second and third binding events are cooperative if *K*_2_ > *K*_1_^2^/3, and if *K*_3_ > *K*_2_*K*_1_/3, respectively.

Given that the three DDRs of the *bind* locus constitute identical binding sites, as shown in the Results, and considering the case of three independent sites (i.e. there is no cooperative binding), the following expressions are obeyed:

$$\begin{equation*}{\rm{\ }}{k}_{12} = {k}_{13}\ = {k}_{23}\ = \ 1\end{equation*}$$

${\rm{\ }}{k}_1 = {k}_2\ = {k}_3$ , which are designated as *K*_a_, or association constant to a DDR

Therefore, as long as this premise is fulfilled, it is possible to determine the association constant *K*_a_ to a single DDR by knowing the macroscopic equilibrium constants calculated in the cooperativity assays. By making substitutions in Equations 2b–2d:

(3a) $$\begin{equation*}{K}_1 = \ 3\ {k}_1 = {\rm{\ }}3K{\rm{a}}\end{equation*}$$

(3b) $$\begin{equation*}{K}_2 = \ 3\ k_1^2 = {\rm{\ }}3K_{\rm{a}}^2\end{equation*}$$

(3c) $$\begin{equation*}{K}_3 = \ 3\ k_1^3 = {\rm{\ }}3K_{\rm{a}}^3\end{equation*}$$

Using Equations 3a–3c, an average value of *K*_a_ was calculated from the macroscopic constants obtained in several EMSA assays.

### Nicking and strand-transfer experiments

For cleavage and strand-transfer assays, 2.5 pmol 3′-Cy5-labelled 27-mer oligo substrate containing the nick sequence was mixed with 25 pmol unlabelled 30-mer oligo (Sigma Aldrich; Supplementary Table S1), which provided the 3′-OH substrate for strand transfer, thus avoiding the re-joining of the 27-mer oligo. The mixture was incubated for 1 min at 37°C with 0.25 pmol of OBD (wt or mutants) in 10 μl of buffer B supplemented with 1 mM MnCl_2_. Protein samples were previously diluted in 20 mM Tris–HCl (pH 8.0) supplemented with 430 mM NaCl and 0.2 mg/ml BSA. After incubation for 1 min at 37°C, the reaction mixtures were treated with proteinase K (60 μg/ml; Roche, 3115879001) and 0.05% SDS for 10 min at 37°C. Before electrophoresis, the samples were mixed with 10 x DNA loading buffer (10 mM EDTA and 60% glycerol) and denatured for 3 min at 95°C. The products were separated on 20% PAA (19:1 acrylamide:bis-acrylamide; BIO-RAD, 1610144), 8 M urea denaturing gels. After electrophoresis, the gels were analysed using a FLA-3000 (FUJIFILM) imaging system, and the reaction products were quantified using QuantityOne software (BIO-RAD).

### Footprinting experiments

Binding reactions for footprinting assays were performed in buffer B containing 6 μM OBD or 0.6 μM His-OBD, and 20 nM of the 3DDR oligo (Supplementary Table S1) labelled at both 5′-ends with ^32^P. Binding mixtures were incubated 20 min at 25°C, and HO• and DMS footprinting reactions were performed as described (9).

### Surface plasmon resonance (SPR) experiments

Standard SPR analyses using a BIAcore 2000 instrument (Cytiva) were performed following the manufacturer's instructions. A streptavidin matrix-coated SA sensor chip (Cytiva, BR100398) was used during DNA–protein interaction analysis. 5′-Biotinylated DDRC or NS dsDNA oligos (Supplementary Table S1) were immobilised on the chip surface to yield a final value of ∼100 RU. In all SPR experiments, HBS-EP (10 mM HEPES, pH 7.4, 150 mM NaCl, 3 mM EDTA, 0.005% Surfactant P20) was used as running buffer. Buffer flow was set to 10 μl/min and each injection had a volume of 30 μl. The surface of sensors was regenerated using 5 μl 0.1% SDS. The results are presented as sensograms obtained after subtraction of the background response signal acquired in control experiments with buffer injections. Triplicate experiments were performed for each OBD–dsDNA interaction analysed.

### Crystallisation of RepB

Full-length RepB was purified as previously described (9). Crystals were obtained by a hanging-drop vapour-diffusion method in the presence of 100 mM Na/K phosphate pH 6.2, 200 mM NaCl and 10% PEG 8000. Crystals belong to the monoclinic space group *P*2_1_ and diffract to 2.77 Å resolution (Supplementary Table S2).

### Crystallisation of the OBD domain

His-OBD was purified as previously described (9), and crystals were obtained by a hanging-drop vapour-diffusion method, in the presence of an equimolar concentration of the ssDNA Nick oligo (Biomers; Supplementary Table S1), in 0.1 M Tri-sodium citrate pH 5.0, 1 M LiCl, and 30% PEG 6000. Crystals belong to the orthorhombic space group *P*2_1_2_1_2_1_ and diffract to 1.50 Å resolution (Supplementary Table S2).

### Crystallisation of the OBD–DNA complex

A 23-bp dsDNA (23AB) was designed, including the DDRI and DDRII sequences from the plasmid pMV158 *bind* locus (9). Synthetic oligos 23A and 23B (Biomers; Supplementary Table S1) were annealed at 80°C for 10 min and then left to cool slowly o/n. The complex of His-OBD with DNA was prepared by incubating the OBD with dsDNA 23AB in a 2:1 ratio in buffer containing 20 mM Tris–HCl pH 8.0, 250 mM NaCl, 1 mM EDTA, 5 mM DTT and 5% ethylenglycol. Crystals of OBD-23AB were obtained by a sitting-drop vapour-diffusion method in the presence of 50 mM Tris–HCl pH 8.0, 50 mM MgCl_2_ and 23% PEG3350. Crystals belong to the monoclinic space group *P*2_1_ and diffract to 3.0 Å resolution (Supplementary Table S2).

### Data collection and structure determination

The diffraction data were collected using the XALOC beamline at the ALBA synchrotron (Cerdanyola del Vallès, Spain (11)) and ID30A-3 beamline at the ESRF synchrotron (Grenoble, France). The structures were solved by molecular replacement with PHASER (12), using one of the full-length RepB protomers or the OBD, from the two structures available to date (accession numbers 3DKX and 3DKY at the Protein Data Bank) as a starting search model (4). For the OBD–DNA complex, the DNA structure was manually modelled base by base using Coot (13), since the electron density map was of enough quality to build the sequence and the entire oligonucleotide was clearly visible. All structures were refined with Refmac5 (14) within CCP4i (15) and Phenix suites (16), applying non-crystallographic symmetry when necessary, and Coot (Supplementary Table S2). DNA parameters were calculated with 3DNA (17). Protein secondary structure elements were defined using DSSP (18). The |*F*_o_| – |*F*_c_| omit maps (Supplementary Figure S7) were calculated for the OBD residues and the bases they interact with, with Phenix (16).

## RESULTS

### New RepB hexamer crystal structure

**Figure 1.**
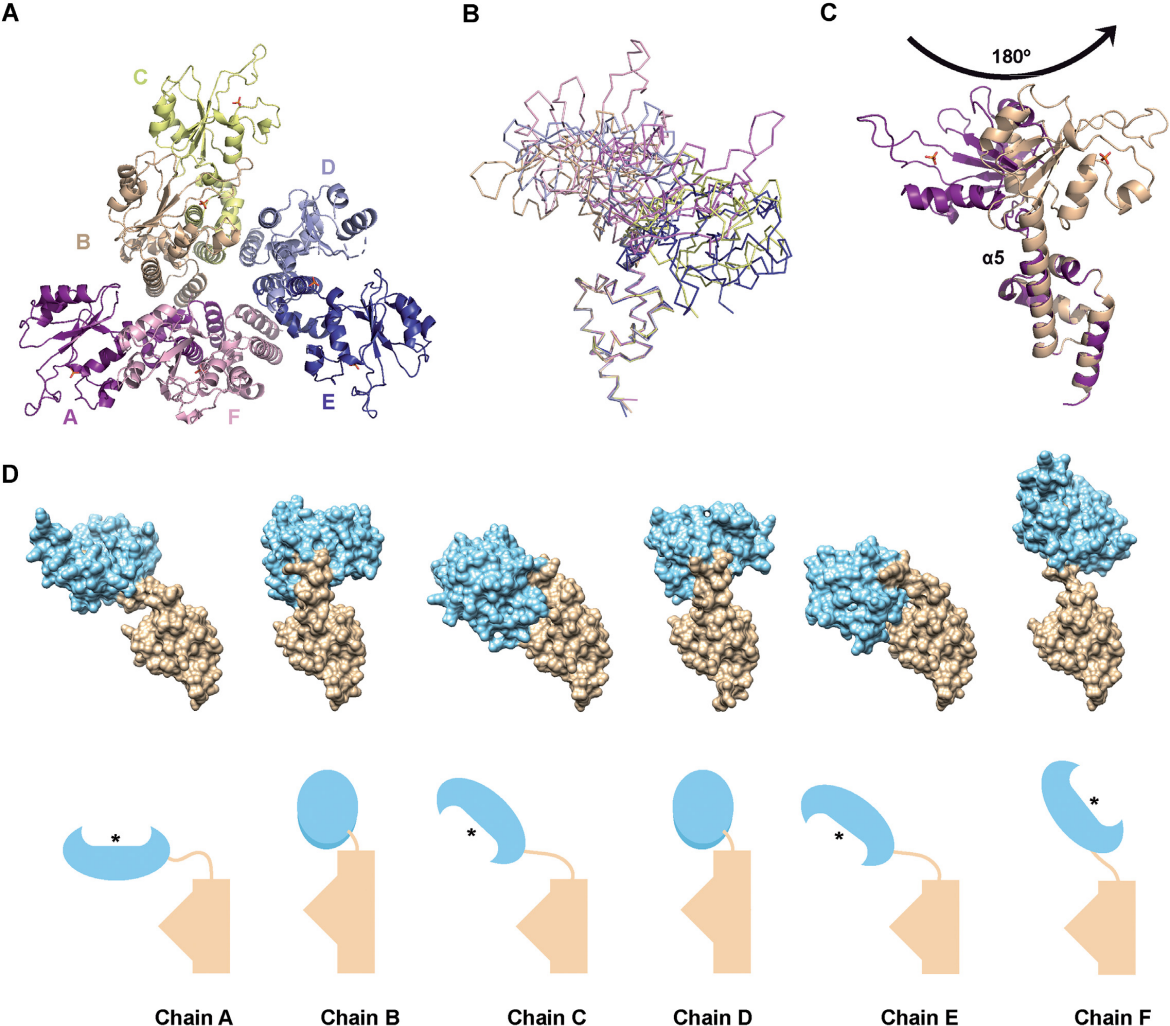
New RepB hexameric structure. (**A**) Cartoon representation of the new RepB_6_ structure, viewed from the OBD. Each protomer is represented by a different colour (chain A in deep purple, chain B in wheat, chain C in pale yellow, chain D in light blue, chain E in deep blue and chain F in light pink). The missing residues in protomer D are represented by a dotted line connecting the rest of the structure. (**B**) Ribbon illustration of the superposition of the ODs of all six protomers of the new RepB_6_, showing the different orientations of the OBD with respect to the OD. Each subunit is indicated by a different colour as in (A). (**C**) Cartoon representation of the superposition of protomer A (purple) and B (wheat) of the new RepB_6_ by the ODs, showing the different orientations of the OBDs with respect to their ODs caused by the presence of an elongated α5 helix in monomer B, connecting the two domains. A phosphate molecule is symbolised in the active site, using a ball-and-stick representation. (**D**) Surface representation (top) and schematic representation (bottom) of the orientation of the OBD (sky blue) relative to the OD (tan) for each monomer within the new RepB_6_. All the monomers are displayed to show the same orientation of the OD. The asterisk indicates the position of the active site.

A new crystal structure for the RepB hexamer was obtained without DNA in the crystallisation conditions (Supplementary Table S2). This structure displays the same six-fold symmetry hexameric ring as a result of the identical disposition of the C-terminal ODs, which show a root mean squared deviation (r.m.s.d.) of the C_α_ positions of 0.318 and 0.497 Å, compared to the OD of the RepB structures 3DKX and 3DKY, respectively. This new structure differs from the previous two in the organisation of the OBDs within the hexamer, which are arranged as a trimer of dimers, in a near three-fold symmetry, and in a distinct orientation of the OBD domains compared to the 3DKY structure (Figure 1A and Supplementary Figure S1A).

The interdomain flexibility has already been described in (4,19), where up to nine different arrangements of the OBD relative to the OD are depicted. Considering this new structure, another six additional orientations should be included (Figure 1B and D). Thus, protomers A and F within this new structure show the closest conformation to the previously described monomers within 3DKX and 3DKY structures (4,19). However, the most striking feature of this new structure is the prolongation of α5 helix by two turns, expanding to the region connecting the ODs and OBDs, in two of the protomers (chains B and D), in place of the 3_10_-helix and the flexible region (residues 128–153) observed in the previous hexameric structures and in the remaining protomers of the current structure (Figure 1C and Supplementary Figure S2D). The different lengths of the α5 helix do not seem to be a consequence of the crystal packing, since there are no crystal contacts in this area. Alignment through the OD region of protomers with this extended helix (chains B or D) against a protomer with a flexible region (i.e. chain A) shows a large anticlockwise rotation of the OBD of almost 180° relative to the OD (Figure 1C). As a result of this new arrangement, the OBD of these two chains is positioned over the OBD of the neighbouring protomers, chains C and E, respectively, thereby occluding access to the active site of protomers B and D within the structure and impeding the cleavage activity on the nick site (Supplementary Figure S2). Therefore, these two protomers must be inactive within this hexamer conformation.

Another consequence of the presence of a longer α5 helix in two of the monomers is that the neighbouring subunits (protomers C and E) also present a dramatic change of orientation of the OBD relative to the OD. Since the OBD of chains B and D are in such immediacy to those of subunits C and E, respectively, the active sites of the latter are forced to move closer to the OD and face it, instead of being exposed at the top of the cup-shaped hexamer (Supplementary Figure S2).

The new layout of the OBDs resulting from the extended α5 helix also influences the positioning of the α2 helix, responsible for the binding of the OBD to the DDR region of the plasmid *dso* (see details below), in four out of the six protomers. The α2 helix faces the outer circumference of the hexamer in subunits B, C, D and E, instead of being exposed at the top of the cup, as in the case of the other two chains, A and F, and in all the protomers of the two structures previously reported (Supplementary Figure S1B and (4)). In this outer orientation, the α2 helix is fully exposed, and in the appropriate position to interact with the DNA major groove of the DDRs (see below).

A phosphate molecule bound to the active site is observed in all the subunits of this new hexamer, instead of the catalytic Mn^2+^ metal ion reported in the other two hexamers. The presence of this phosphate may be explained by the crystallisation conditions, which included Na/K phosphate buffer at pH 6.2, and the lack of Mn^2+^ or any equivalent divalent metal ion, either during the purification of the protein or in the crystallisation drop. An alignment of the C_α_ positions of one of the monomers within this structure, through the OBD, with one of the monomers of the 3DKX structure previously reported, shows that this phosphate molecule is between 1.8 and 2.4 Å away from the position of the Mn^2+^ and, consequently, from the histidine triad that coordinates it in the previous structures. Furthermore, the phosphate molecule establishes interactions with additional residues to those coordinating the Mn^2+^, such as Tyr99, His102 and Lys52. The presence of a molecule of phosphate instead of the catalytic metal ion located in the active site at a short distance from the position of the Mn^2+^ metal ion and the triad of histidines has already been reported for TrwC protein, another member of the HUH family, more precisely of the relaxase group (20).

### Structure of the OBD alone

A very high-resolution 1.50 Å structure of the OBD alone was obtained (Supplementary Table S2). Although the crystals grew in the presence of ssDNA of the nick sequence, there was no DNA bound to the protein. There is only one molecule of the OBD in the asymmetric unit, which displays a folding similar to the OBDs of the previously described 3DKX and 3DKY hexamers, and of the hexamer currently described here, with a r.m.s.d. of the C_α_ positions that ranged between 0.552 and 0.677 Å, depending on the OBD considered. The main differences observed are located on flexible regions, such as the loop between β-strands β2–β3, which includes the residues coordinating the Mn^2+^ metal ion (data not shown).

### Characterisation of OBD binding to the DDR sequences

**Figure 2.**
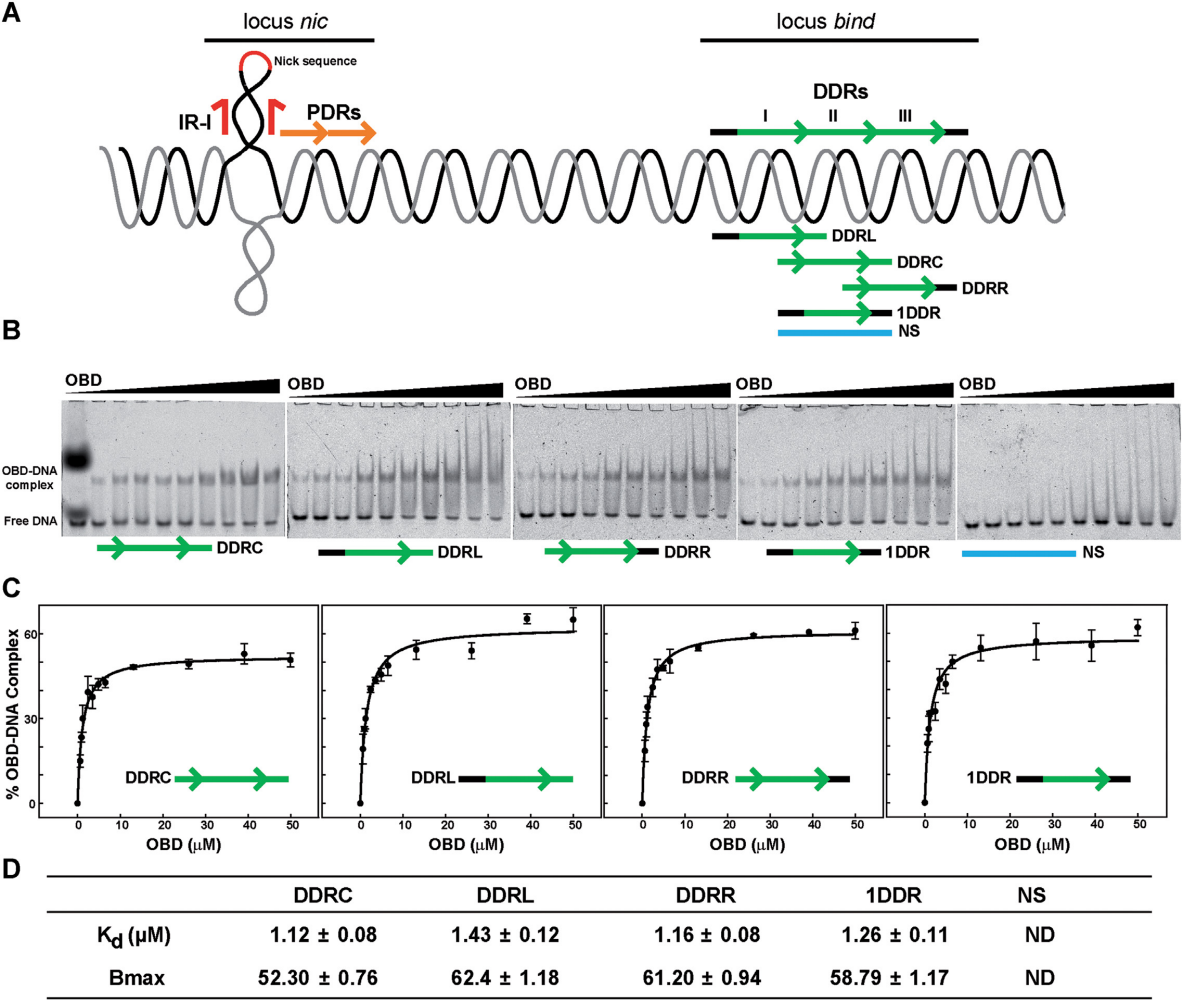
Binding affinity of the OBD to the three DDR sequences of the *bind* locus. (**A**) Schematic representation of the pMV158 *dso*, showing the approximate relative locations of the nick sequence, the proximal direct repeats (PDRs) of the *nic* locus, and the distal direct repeats (DDRs) forming the *bind* locus. A schematic representation of the 19-bp dsDNA oligos used in the binding affinity assays is shown below the DDR sequence. (**B**) EMSA using increasing concentrations of the OBD (0, 0.6, 0.9, 1.2, 2.4, 3.5, 5.0, 6.5, 26.1, 39.1 and 49.9 μM) and 20 nM of the different DNAs depicted in (A), fluorescently labelled. The first lane of the gel showing the EMSA with DDRC corresponds to the protein-free DNA sample. In this lane it is also visible the fluorescence emitted by bromophenol blue and xylene cyanol tracking dyes. This gel does not include the binding reaction containig 2.4 μM of OBD, although it was incorporated in the replicate gels. Gels display representative EMSA from at least three independent experiments that were performed for the *K*_d_ and *B*_max_ determination as depicted in (D). (**C**) The graphs show the plot of the percentage of OBD–DNA complex as a function of the concentration of the OBD, obtained from the EMSA experiments in (B). Symbols represent the average value of three independent assays, and error bars show the standard deviations. Curves were fitted by nonlinear regression (solid lines) to a ligand binding model assuming a single class of binding site for DNA. (**D**) The table contains the best-fit values (expressed as mean ± standard error) of the apparent dissociation constant (*K*_d_) and of the amplitude of the reaction (*B*_max_) for the interaction between the OBD and the indicated dsDNA oligos. ND refers to not determined.

The binding affinity of the OBD for each of the three DDRs composing the *bind* locus was determined by EMSA (Figure 2). To study the interaction between the OBD and the repeat located in the centre (DDRC), left (DDRL) or right (DDRR) sides of the *bind* locus, all dsDNA fragments used were the same size (19 bp) and included the 11-bp repeat sequence plus the adjacent 4 bp at both ends of each repeat (Figure 2A and Table S1). We also included two additional fragments in the analysis, one (termed 1DDR) with an 11-bp repeat sequence flanked by the sequences adjacent to the entire three-tandem-repeat DDR region, and the other (NS) containing a non-specific sequence. For the EMSA, 20 nM dsDNA oligo was mixed with increasing concentrations of OBD, ranging from 0.5 to 50 μM. The results showed the formation of a unique retarded complex that presumably resulted from the equimolecular interaction between the OBD and the dsDNA fragment (Figure 2B). The exception was the faint smear that appeared with the NS fragment (Figure 2B), which is indicative of weak binding and confirmed the specificity of the interaction between the OBD and the DDR-containing fragments. The apparent binding parameters obtained from these experiments were analysed by one-way ANOVA to account for the differences in the affinity of the OBD for each of the repeats of the *bind* locus. There were no significant differences between the dissociation constant (*K*_d_) values obtained for the interaction between the OBD and the four specific DNA molecules (K_d_ ∼1 μM, p-value of 0.215); therefore, the OBD did not bind preferentially to any of the DDRs of the *bind* locus (Figure 2C and D). We also measured the stability of OBD binding to one repeat of the *bind* locus by analysing the dissociation kinetics of the OBD complexed to fluorescently labelled DDRC upon the addition of a 1000-fold excess of the same unlabelled DNA. Samples were withdrawn at different time intervals and loaded on native PAA gels (not shown). The dissociation rate of the OBD–DDRC complex was very high, since 10 s after the addition of an excess of unlabelled DNA the OBD–DDRC complex was undetectable. Therefore, we conclude that the half-life of the OBD–DDRC complex was less than 10 s.

**Figure 3.**
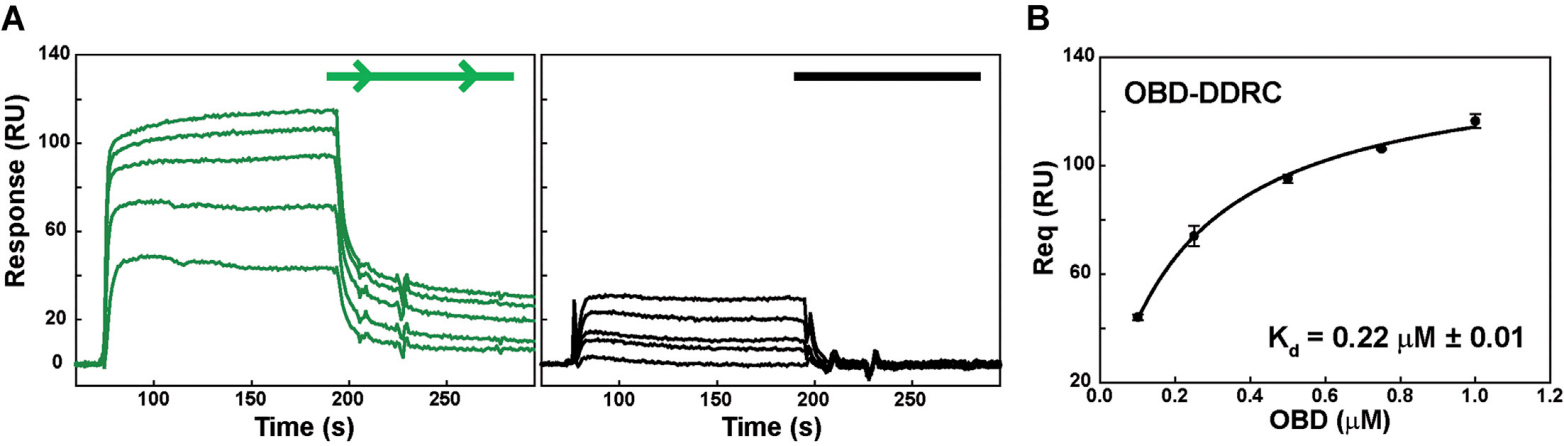
SPR analysis. (**A**) Formation of the complex involving the OBD and dsDNA in real time by SPR, on a streptavidin matrix-coated SA sensor chip with DDRC (green graph) or NS (in black) dsDNAs immobilized on its surface. Purified protein was injected onto the sensor chip at five concentrations: 0.1, 0.25, 0.5, 0.75 and 1 μM. The panel display representative sensograms of one of the three independent experiments performed for the *K*_d_ determination as depicted in (B). (**B**) Steady-state response (Req) obtained from the sensograms was plotted against increasing OBD concentrations (black circles) and fitted to a one-site ligand binding affinity model (solid line) for the determination of the equilibrium dissociation constant (*K*_d_) value. Symbols represent the average value of three independent experiments, and error bars show the standard deviations.

The formation of the complex involving the OBD and DNA sequences of the *bind* locus was also studied in real time by surface plasmon resonance (SPR). Biotinylated 1DDR and NS dsDNA oligos were immobilised on a streptavidin matrix-coated sensor chip and incubated with the OBD. Steady-state response (Req) obtained from the sensograms (Figure 3A) was plotted against OBD concentration (black circles) and fitted to a one-site saturation binding model (solid line) for the determination of the *K*_d_ values (Figure 3B). The binding affinity of the OBD for 1DDR (apparent *K*_d_ = 0.22 ± 0.01 μM) was about 15-fold higher than that for NS (not shown). The *K*_d_ calculated by SPR for the binding of the OBD to 1DDR was of the same order of magnitude as that calculated by EMSA (*K*_d_ = 1.1 ± 0.1 μM), and the differences in the *K*_d_ values obtained by both methods may be attributed to the distinct experimental conditions under which the assays were performed (see Materials and Methods).

EMSA analysis of the binding of the OBD to a 42-bp dsDNA oligo (3DDR) that contains all three DDRs of the *bind* locus revealed the generation of three complexes (C1, C2 and C3), which were differentially populated at each protein concentration tested (Supplementary Figure S3). At the lowest OBD concentrations, only the fastest-migrating C1 was detected. At higher protein concentrations, C1 decreased while complexes C2 and C3 sequentially appeared and accumulated. The observed pattern of complexes is compatible with C1 resulting from the binding of the OBD to a single repeat, C2 from binding of the protein to 2 repeats, and the slowest-migrating C3 arising from the saturation of the three identical sites of the 3DDR dsDNA (Supplementary Figure S3). Potential cooperativity in the binding of monomeric OBD to the three DDRs of the *bind* locus was addressed by analysing the relative amount of the different complexes formed at various protein concentrations, which allowed estimation of the macroscopic constants for binding to one, two, or all three sites ((10), see Materials and Methods). The values of the ratios of *K*_2_ to *K*_1_^2^/3 (1.50 ± 0.52) and of *K*_3_ to *K*_2_*K*_1_/3 (0.71 ± 0.25) do not allow us to infer cooperativity in the second and third protein binding events that would result, respectively, in the generation of C2 and C3. The binding to identical (same intrinsic affinity) sites is the condition that best exposes cooperativity of binding (10), therefore the values obtained rather indicate independent binding of the OBD to the three identical DDRs composing the *bind* locus of the plasmid *dso*. Under this premise, an average value of 1.2 ± 0.4 μM^−1^ could be obtained for the association constant (*K*_a_) from the macroscopic constants *K*_1_, *K*_2_ and *K*_3_ estimated by EMSA (see Materials and Methods). This gives a *K*_d_ (1/*K*_a_) of ∼ 0.8 μM for the binding of the OBD to any of the DDRs, in good agreement with the dissociation constant values determined for the binding of the protein to the separate DDRs (Figure 2D).

### Analysis of the residues involved in the OBD–DDR interaction

**Figure 4.**
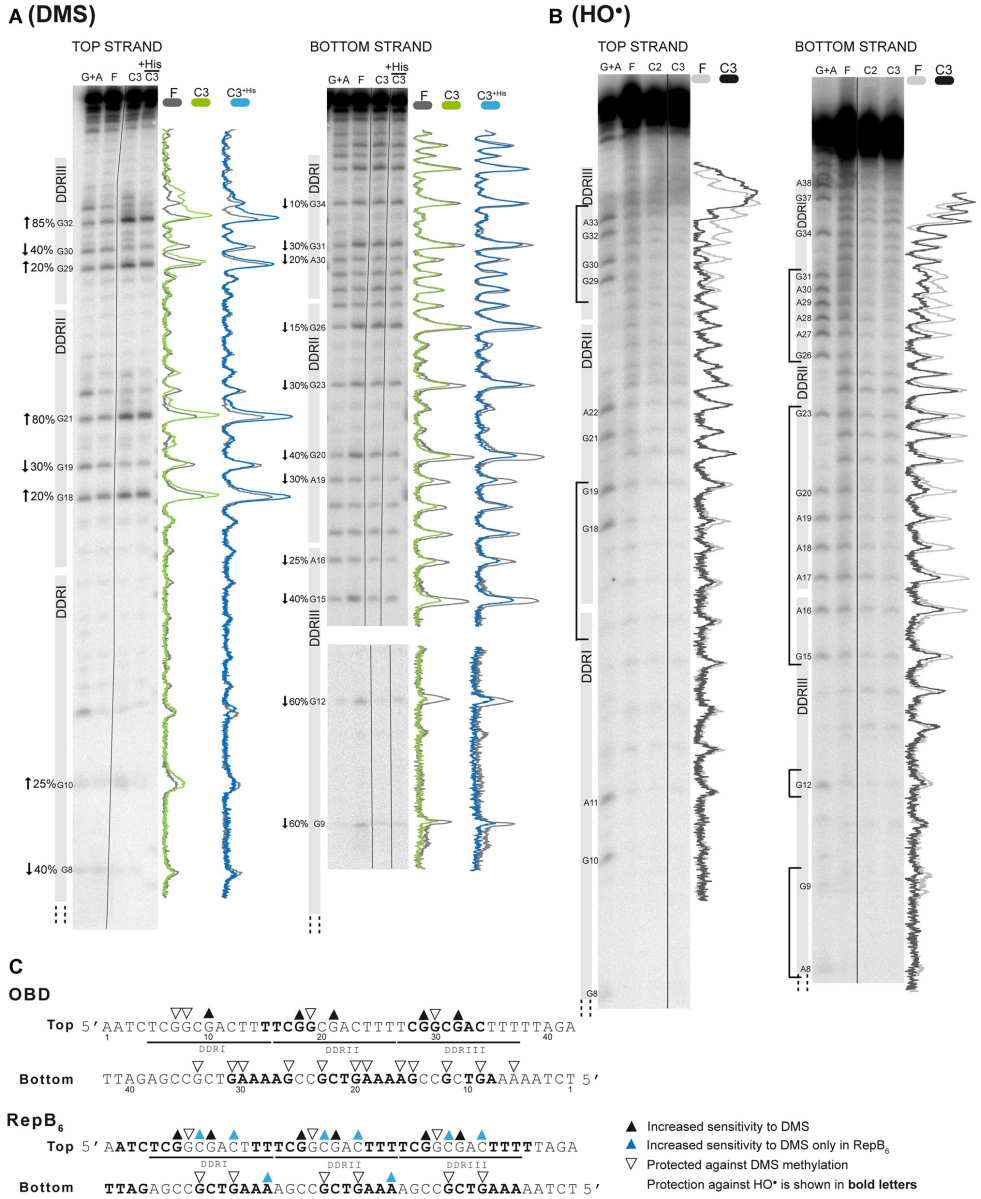
High-resolution contacts of the OBD with the *bind* locus. (**A**) DMS footprinting of the OBD (±His-tag) bound to a dsDNA oligo containing the entire sequence of the *bind* locus (3DDR). Sequencing gels show the methylation patterns of the two strands after treatment with DMS. Absorption scans of naked DNAs (grey lines) and C3 complexes formed with the OBD (green lines) or with His-OBD (blue lines) are shown to the right of the corresponding gel. To the left, the percentage of hyperexposure (upwards arrows) or protection (downward arrows) of the bases with an altered methylation pattern is indicated. The value given in the figure is the average percentage calculated for each base from the analysis of at least two different sequencing gels. Lanes: G + A, Maxam and Gilbert sequencing ladder; F, naked DNA; and C3, DNA of the C3 complex. Images from the same gel were grouped and are indicated by a dividing line. In the analysis of the bottom strand, images of two sequencing gels of the same strand were combined and are displayed together, the largest one showing up to G15 position and another showing from G12 to G9. (**B**) The 5'-end labelled 3DDR dsDNA was incubated with the OBD and subjected to treatment with HO•, as depicted in Materials and Methods. Sequencing gels show the modification patterns of the two strands. Absorption scans of naked DNAs (light grey lines) and C3 complex (dark grey lines) formed between the OBD and 3DDR are shown to the right of the corresponding gel. To the left, the sequence regions protected against the action of HO• are indicated with brackets. Lanes: G + A, Maxam and Gilbert sequencing ladder with the position of the purines indicated; F, naked DNA; and C2 and C3, DNAs of the C2 and C3 complexes. Images from the same gel were grouped and are indicated by a dividing line. (**C**) Summary of the contacts established between the OBD and the *bind* locus, compared to those previously identified for RepB_6_. Bases hyperexposed (black triangles) or protected (open triangles) by the OBD or RepB_6_ against methylation with DMS are shown. Bases that become hypermethylated only upon binding of RepB_6_ are indicated by blue triangles. Bases whose deoxyriboses are protected by the OBD or RepB_6_ from HO• cleavage are shown in boldface letters.

Contacts established by the OBD with the DNA of the *bind* locus were analysed by HO• and DMS high-resolution footprinting experiments of the separate nucleoprotein complexes generated by binding of the protein to the 42-bp 3DDR DNA. To test whether the presence of the His-tag in the N-terminal end of the OBD altered the interaction with the dsDNA, we performed footprinting analysis with both versions of the protein, that is to say, with and without the His-tag. Although protein concentrations giving rise to C1, C2 and C3 were used in these assays, the yield of C1 was always insufficient to allow its characterisation. Moreover, due to the short length of the 3DDR DNA, the reactivity to DMS or HO• of the nucleotides located close to the ends of the top and bottom strands could not be analysed properly. Nevertheless, the results showed changes in the DMS reactivity profiles of the 3DDR DNA upon OBD binding, with a clear repeated methylation pattern. The OBD interacts through the major groove of the DNA with the same three Gs of each repeat (the top strand G8 and the bottom strand G31 and G34 in DDRI, the top strand G19 and the bottom strand G20 and G23 in DDRII, and the top strand G30 and the bottom G9 and G12 in DDRIII), protecting them against methylation (Figure 4A and C). Additionally, the bottom strand G26 in DDRII and G15 in DDRIII were protected against methylation. We also observed enhanced reactivity to DMS in particular Gs of each repeat (G10 in DDRI, G18 and G21 in DDRII, and G29 and G32 in DDRIII). Since no hydrophobic residues appear close to these Gs in the crystal structure of the OBD–DNA complex (see below), the observed hyper-methylation might arise from the increased accessibility of those bases to DMS as a result of protein-induced distortion of the DNA double helix (9,21). Analysis of the DNA helical parameters (17) in the protein-DNA complex crystal structure (see below) shows that some of the base-pairs with enhanced G reactivity (G10, G21) have a high buckle or high stagger. Whether these distortions cause the enhanced reactivity is unclear, since other bases (G18) do not show them, but they are hypermethylated. Overall, the DMS reactivity pattern at the G level was identical in the C2 (not shown) and C3, irrespective of the presence of the His-tag (Figure 4A), and roughly matched that observed for the interaction of RepB_6_ with the same DNA sequence (Figure 4C; (9)). However, differences between the methylation patterns induced by the full-length protein and the OBD were observed in some Cs located on the top strand of each repeat, whose DMS-reactivity was enhanced only in the complexes formed by RepB_6_ (Figure 4C). According to these results, the binding of RepB_6_, but not the OBD, to the *bind* locus could cause the severe distortion of the DNA double helix that is required for these C residues to become susceptible to methylation. Protection against HO• cleavage showed that the three DDRs were bound by the OBD, although the footprints showed fewer contacts with the DNA backbone of the DDRI and DDRIII sequences in comparison with those observed for RepB_6_ when interacting with the same sequence (Figure 4B and C).

### Crystal structure of the OBD–DNA complex

**Figure 5.**
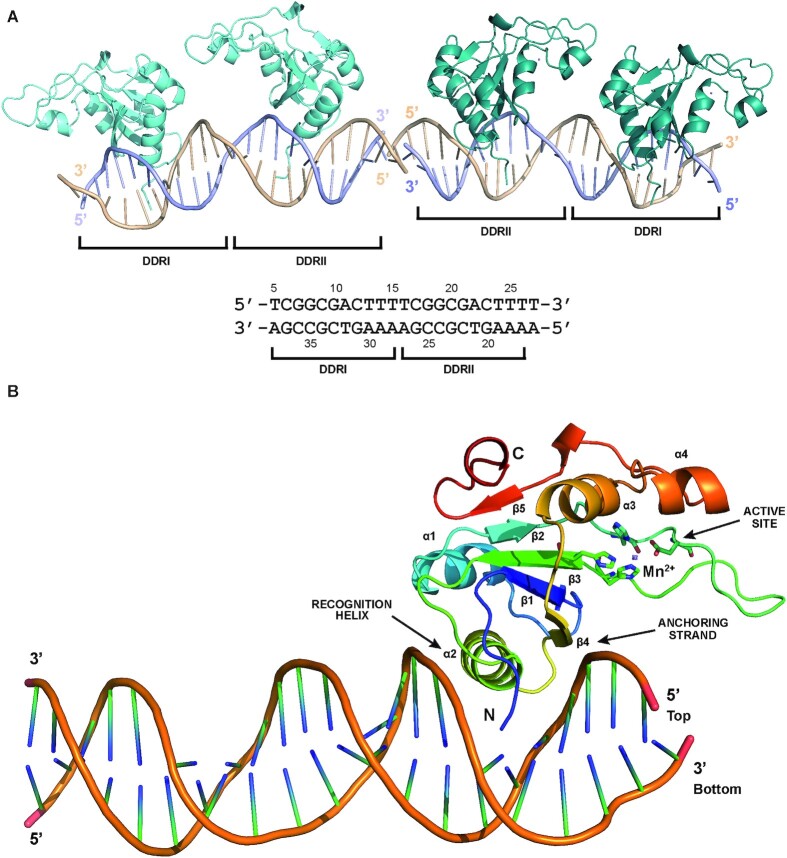
Structure of the OBD–DDR complex. (**A**) Ribbon representation of the OBD–DDR complex present in the asymmetric unit, containing two molecules of the dsDNA 23AB, oriented in opposite directions, and two molecules of OBD bound to each dsDNA (in deep teal and aquamarine colours, respectively). Each strand of DNA is pictured by ribbon in light blue (top strand) and wheat (bottom strand). The sequence of the dsDNA 23AB is depicted underneath the structure. The DDRI and DDRII regions are delimited both in the structure and the sequence. The numbering indicated above and below the sequences corresponds to the position of the nucleotide within the DDR, based on the numbering displayed in Figure 4C. (**B**) Close-up view of one of the OBDs portrayed in ribbon representation bound to DNA, showing the different structural elements involved in DNA binding in rainbow colours, including the recognition helix and the anchoring strand. The active site of the domain, with the residues involved in the coordination of the Mn^2+^ ion are shown in ball-and-stick representation.

The OBD was crystallised in complex with a 23-bp dsDNA fragment (23AB) containing two out of the three repeats from the *bind* locus (Supplementary Table S2). In this complex, the asymmetric unit consisted of two molecules of the DNA oriented in opposite directions, and two molecules of OBD bound to each DNA molecule (Figure 5A). The DNA molecules are in B conformation, with an estimated curvature of the DNA of ∼25°. The two OBDs are positioned on the same side of the DNA molecule because they are all bound to the GC-rich region within the 11-bp tandem direct repeats, which are separated by a complete double helix turn. The binding to the GC-rich region in the absence of DNA bending causes the lack of protein-protein interactions between the OBDs bound on the same DNA molecule (Figure 5A), in agreement with our results indicating that there was no cooperative binding of the OBD to the DNA (see above).

The global fold of the OBDs bound to the DDRs is identical to the OBD in the stand-alone structure described above, with a r.m.s.d. of the C_α_ positions of 0.64 Å. However, alignment of these two structures reveals clear differences located in the flexible loop connecting β2 and β3 β-strands and in the loop connecting α2 and β4, due to its proximity to the DNA. The binding of the OBD to the DDR drives this latter loop to move 3.16 Å away so that Met86 locates at approximately 3.0 Å of G8 of the top strand in the OBD–DDR structure (Supplementary Figure S4). This movement includes the 180° flip of the Ala85-Met86 peptide bond and results in the extension of strand β4 by four residues in the N-term direction (Supplementary Figure S4).

All OBD molecules in the asymmetric unit interact with the DDRs in the same way (Supplementary Figure S5). The interaction of the OBD with the DNA of the *bind* locus occurs through the major groove and involves residues situated in the N-terminal part of the domain, namely the N-terminal tail, the α2 helix, and the neighbouring loops connecting to β3, α2 and β4, and the extended strand β4 or anchoring strand (Figure 5B).

**Figure 6.**
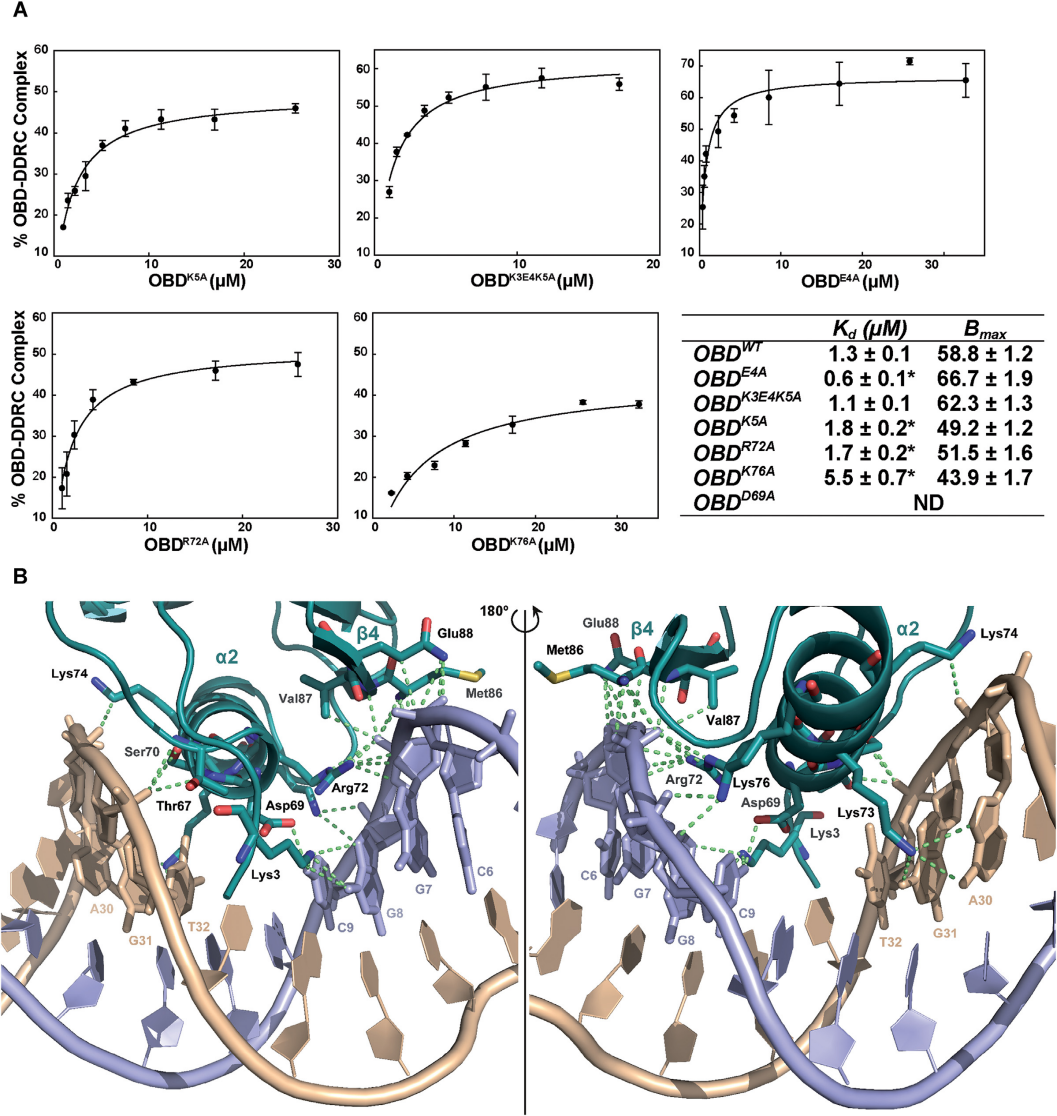
DDR binding affinities of the OBD mutants and detailed OBD–DNA contacts. (**A**) The graphs show the plot of the percentage of OBD–DNA complex as a function of OBD concentration, obtained from the EMSA experiments for the OBD mutants Lys5Ala, the triple mutant Lys3, Glu4 and Lys5 to Ala, Glu4Ala, Arg72Ala and Lys76Ala. Symbols represent the average value of three independent EMSA assays, and error bars show the standard deviations. Curves were fitted by nonlinear regression (solid lines) to a ligand binding model assuming a single class of binding site for DNA. The table contains the best fit values for the apparent dissociation constant (*K*_d_) and for the amplitude of the reaction (*B*_max_) for the interaction between the different OBD mutants and the dsDNA oligo 1DDR. Differences among *K*_d_ values were analysed by Student's *t*-test. The asterisks indicate statistically significant differences (*P* < 0.05) with the K_d_ value of the OBD^wt^. ND denotes not determined. (**B**) Detail of the crystal structure of the DNA-protein interface of the OBD–DDR complex. The OBD (chain A) is represented by ribbon in deep teal, with the highlighted residues involved in the interaction with the DNA in ball-and-stick, and the interactions established with the DNA in lime colour dashes. Each strand of DNA is pictured by ribbon in light blue (top strand) and wheat colour (bottom strand), respectively, with the highlighted nucleotides in ball-and-stick, and numbering the bases as in Figure 5A. The image on the right shows a 180° rotated view of the image on the left.

The N-terminal tail enters the DNA major groove at Lys3, whose side chain Nζ makes an H-bond to O6 of G8 and G19 at the top strand of DDRI and DDRII, respectively (Figure 6B, Supplementary Figures S6 and S7). This observation is consistent with the footprinting results shown above, where there is methylation protection of the two bases upon OBD binding (Figure 4C). The next lysine residue in the sequence, Lys5, also contacts the DNA at a backbone phosphate.

More extensive are the contacts of the α2 helix, which fully penetrates the major groove, adjacent to where the N-terminal tail is situated. Specific contacts include H-bonds between the carboxyl group of the side chain of Asp69 and the N4 of the top DNA strand C9 and C20, at DDRI and DDRII respectively, and between the Nζ of Lys73 and the O6 and N7 atoms of the bottom strand G20 and G31, at DDRI and DDRII, respectively (Figure 6B, Supplementary Figure S6 and S7). The presence of these interactions is supported by the footprinting results shown earlier, where there is methylation protection of those two guanine residues upon OBD binding (Figure 4C). The Cβ atom of Asp69 and the Cα atom of Ser70 create a hydrophobic pocket that allocates the 5-methyl group of the bottom strand T21 and T32 at DDRI and DDRII, respectively.

Other interactions are non-specific, and occur with the DNA backbone phosphates, involving Ser70, Arg72, Lys73, Lys74 and Lys76, all in α2 (Figure S6). Further non-specific interactions with backbone phosphates include Thr67 at loop β3-α2 and a double H-bond between the amide nitrogen atoms of Met86 and Val87 to two phosphate oxygen atoms of C8 and C19. These two protein residues belong to the extended part of β4 that is organised as such upon DNA binding. The β4 strand is at the edge of the central β-sheet of the OBD and is thus available for binding the DNA as it runs parallel to the DNA backbone.

The involvement of some of these residues in the binding to the DNA has been proposed previously, as the mutation of Arg72 or Lys76 to Ala affects the binding capacity of these mutant proteins to the *bind* locus, whilst the quadruple mutant Arg72, Lys73, Lys74 and Lys76 to Ala is unable to bind to it (4).

### OBD mutagenesis

To analyse the role of several residues of the OBD in the binding affinity for the DDR sequence, another set of mutations was designed. Considering the amino acids involved in the interaction of α2 with the DNA in the crystallographic complex structure, OBD mutants with substitution of Asp69, Lys73 or Lys74 to Ala were designed to test their binding affinity to the 1DDR oligo. Although several trials were attempted, the introduction of an Ala residue instead of Lys at positions 73 or 74 could not be accomplished, thereby precluding the characterisation of the activity of the corresponding mutants. In contrast, the OBD^D69A^ mutant was constructed, purified following the same protocol as described before for the OBD^wt^ (4), and tested by EMSA for binding to the 1DDR oligo. The DNA binding ability of the OBD^D69A^ mutant was analysed and compared to the results obtained with OBD^R72A^, OBD^K76A^, and the mutant with the quadruple substitution Arg72Ala, Lys73Ala, Lys74Ala and Lys76Ala. OBD^D69A^ bound to 1DDR with much lower affinity than OBD^wt^ and did not form a stable complex, as suggested by the appearance of a smear instead of a defined band in EMSA (data not shown), thus preventing the determination of the binding affinity constant for this mutant. On the other hand, OBD^K76A^ showed a dissociation constant around five times higher than OBD^wt^ (*K*_d_ = 5.5 ± 0.7 μM; Figure 6A), while the quadruple mutant did not bind to the DNA (data not shown). Finally, the OBD^R72A^ mutant was affected the least, with a binding affinity for the DNA similar to that of OBD^wt^ (*K*_d_ = 1.7 ± 0.2 μM; Figure 6A).

Furthermore, two residues of the N-terminal tail shown in the co-crystal structure, namely Lys3 and Lys5, were found to be involved in the interaction of the OBD with the DDR sequence (Figure 6B). Since these two residues, together with Glu4, are highly conserved among the Rep proteins of the pMV158-plasmid subfamily (Supplementary Figure S8), the three residues were individually changed to Ala. As was the case with Lys73 and Lys74 (see above), the Lys3Ala mutant could not be obtained. Thus, a triple mutant was also designed, replacing the three desired amino acids at once. The mutant proteins were purified and assayed by EMSA for binding to 1DDR (Figure 6A). The OBD^E4A^ mutant unexpectedly showed a two-fold increase in the binding affinity to 1DDR (*K*_d_ = 0.6 ± 0.1 μM; Figure 6A) compared to the wt protein. In contrast, the OBD^K5A^ mutant was able to form a specific complex with the DNA but bound with lower affinity (*K*_d_ = 1.8 ± 0.2 μM) (Figure 6A), whereas the triple mutant Lys3Ala, Glu4Ala and Lys5Ala bound to the DNA with the same affinity as OBD^wt^ (*K*_d_ = 1.1 ± 0.1 μM). In this latter case, the improvement in binding observed by the Glu4Ala change was counteracted by the introduction of the other two mutations, but neither had a dramatic effect on the DNA binding capacity of these mutants. Therefore, the N-term end of RepB is unlikely to have a critical role in binding to the DDR, due to its flexibility and the high B-factors in all the OBD structures in the crystal, but it supports the main anchoring of RepB to the G/C bases of the DDR through the α2 helix. It is important to mention that none of the mutants described herein, or the previously designed substitutions (namely Arg72Ala, Lys76Ala and the quadruple mutant Arg72, Lys73, Lys74 and Lys76 for Ala (4)) were affected in the nicking and strand-transfer activities compared to OBD^wt^ (Supplementary Figure S9). These results indicate that RepB not only can be divided into two structural and functional domains (OBD and OD) as described so far, but two distinct regions within the OBD have independent activities, namely, the DDR specific recognition, localised mainly at the α2 helix, which we name the recognition helix, and the nicking and strand-transfer catalytic activities on the nick sequence, which involve residues located in the loop between β2 and β3 strands, in strand β3 and the α3 helix.

## DISCUSSION

Here, we solve a new structure of full-length RepB_6_ and reveal the high structural versatility of this protein. There are three different snapshots of the possible conformations of the hexameric form, which include 15 alternative dispositions of the OBD relative to the OD within the same protomer. All these conformations have in common that the ODs arrange on a near 6-fold symmetry, with small fluctuations between the three structures, while the OBDs are arrayed either on a two-fold or a near three-fold symmetry, with very high variability in the conformation adopted.

The most remarkable characteristic of the new structure depicted here is the presence of an extended α5 helix in two of the protomers (B and D) in the region connecting the two domains, instead of a flexible region. The first implication of this new conformation is the lack of flexibility in the interdomain region, which would limit the freedom of movement of these OBDs. More importantly, it may also affect the activity of these two protomers, since their OBD is placed on top of that of the adjacent subunit. Consequently, the active site is hidden within the structure of the hexamer. Hence, these two protomers are unable to perform the cleavage reaction while in this conformation. On the other hand, the partnering protomers (C and E) of those with the extended α5 helix are also subjected to a dramatic conformational change. The position of the neighbouring OBD above them pushes their active site closer to the OD, with their active sites ‘down’, that is 180° away from the previously observed structure (3DKX, 3DKY) and from protomers A and F in the present structure. Although the access of the DNA to the catalytic centre may not be fully impeded in the ‘down’ conformation, at least in one protomer (E) appears to be relatively occluded. Therefore, probably only two out of the six existing active sites within the hexamer are accessible for DNA cleavage in the conformation reported herein. It is important to note that only one protomer is required for cleavage activity during the replication initiation step, and the assistance of a second one may be necessary for the second cleavage reaction that takes place during the termination step of the leading-strand synthesis (22,23). Another important feature of the new hexameric structure is the location of the *dso*-recognition α2 helix facing toward the outside of the hexamer in four out of six protomers (B, C, D and E), compared to the originally described structures of RepB, where this helix is on the surface of the cup-like structure in all six protomers. The high interdomain flexibility inferred from the 15 distinct protomer conformations observed in the three solved RepB_6_ crystal structures may enable one hexameric protein to interact simultaneously, through its OBDs, with the distinct elements constituting the *dso* (namely the DDRs of the *bind* locus, and the PDRs and nick sequence of the *nic* locus). Hence, the structural flexibility of RepB might account for the nucleated assembly of what we propose to be the replication initiation active form of the protein (i.e. a ring-shaped hexamer encircling one of the parental DNA strands) upon binding of monomers to the recognition elements of the *dso*. Sequential assembly of functional hexamers or double hexamers of replication initiation proteins upon binding of the monomeric form to specific repeated DNA sequences that constitute the recognition elements of the origin has been previously proposed in several viral systems (24).

The specific recognition sequence of the pMV158 *dso* is the *bind* locus, which constitutes the highest affinity and primary binding site of RepB_6_ (9). Here we have performed a meticulous study of the OBD binding to DDR regions of different lengths from the *bind* locus. To this end, we used EMSA, SPR, footprinting analysis and crystallography to compare the DNA binding properties of full-length RepB_6_ with the separate OBD. The OBD binds with a similar affinity to any 19-bp long dsDNA containing one of the DDRs, irrespective of the surrounding sequences. The OBD–DDR complex is not stable over time, since it disassembles in less than 10 s, when competitor DNA is added to the reaction, compared to the 18 min half-life of the complex between RepB_6_ and the entire *bind* locus (9). We proposed that the high binding stability of RepB_6_ to the 3DDR DNA is due to the presence of the OD, which may help stabilise the complex formed on the DNA as a hexamer, since the OD alone is incapable of binding to DNA (4). Furthermore, footprinting analysis shows that the OBD interacts with the same bases within the DDRs as full-length RepB_6_, although there are some additional cytosines residues exposed to methylation when the latter binds. This result points to severe distortion of the DNA by RepB_6_, a feature that the OBD cannot fully reproduce alone; although a curvature is already observed in the DNA in the OBD–DNA crystal complex reported here. The arrangement of the OBDs in a hexameric form caused by the presence of the OD should force a much higher DNA curvature (estimated to be ∼ 105° (9)) since the OBDs are bound together and cannot interact in tandem in a straight line without describing a pronounced curve. This strong DNA distortion, promoted by the RepB hexamer upon binding, may be necessary for the protein to prompt a radical conformational change in the DNA of the origin of replication that helps to expose IR-I as a cruciform, with the substrate nick sequence in a terminal loop.

Although we were not able to analyse the cooperativity of binding of full-length RepB_6_ to the *bind* locus because of the stability of the hexamer, it was assessed for the OBD, which is purified as a monomer. OBD binding to the entire *bind* locus does not show cooperative behaviour. This observation is consistent with the lack of protein-protein interactions between monomers observed on the crystal structure of the OBD–DDR complex. However, this result does not exclude the possibility of cooperative binding of RepB to the *dso*, as cooperative binding of full-length T antigen (T-ag) protein from SV40 to the *ori* and of Rep68 from AAV2 to the AAVS1 region has also been described, whereas their OBD domains did not show this behaviour (24,25), thereby providing another putative role to the OD of RepB in this matter.

Regarding the structure of the OBD bound to DNA, it is noteworthy that in the OBD–DDR complex, the active site is not found in the vicinity of the DNA. This can be explained by the fact that the DNA fragment used in the crystallisation process was the *bind* locus rather than the *nic* locus, whose nick sequence is specifically recognised and cleaved by the protein active site. The same disposition is also described for the interaction of the Rep protein from adeno-associated virus 5 (AAV5) with the Rep binding site (RBS), constituted by five tandem direct repeats of a tetranucleotide sequence, which is recognised by the surface loop between strands β4-β5 and αC helix (equivalent to α2 of RepB), while the active site of the protein is facing towards the terminal resolution site (trs) to be cleaved (26). Both proteins, together with the replication initiation protein E1 from papillomavirus (27), share an equivalent α helix, as well as β/β or β/α loops, for the recognition of the specific dsDNA sequence to which they bind. However, the recognition mode of the three differs completely (Supplementary Figure S10). Thus, the β4/β5 loop of Rep from AAV5 binds to the major groove of the DNA, whereas the αC helix interacts with bases located on the minor groove (26). In contrast, the OBD of both papillomavirus E1 and pMV158 RepB interact exclusively with bases situated on the major groove of the DNA double helix, although the specific recognition differs considerably in both. In E1 helicase, the recognition takes place through residues located on a DNA binding loop, which contacts the bases of only one of the DNA strands (27), while in RepB, it occurs through the α2 recognition helix fully entering the major groove (Supplementary Figure S10). In RepB, the non-specific binding of β4 strand, namely the anchoring strand, at the edge of the β-sheet, to backbone phosphates, upon enlargement of this secondary structure element of the protein is also unique.

In the recognition helix, the Asp69 is a highly relevant residue for stabilisation of the binding of the OBD to the DDR. It is the only residue that establishes a specific interaction with a cytosine base within the DDR and its replacement by Ala produced a protein that is unable to form a stable complex with this DNA. However, the inability of Asp69Ala mutant to bind to the DDR does not interfere with its capability to perform the cleavage reaction at the nick site within the *nic* locus. These results indicate that the binding and nicking activities in the OBD are located in separate regions, as observed in the complex structure, and that they can be uncoupled by introducing a mutation at any of the residues on α2 helix. These observations thus support the notion that the interaction with the nick sequence can be achieved through another part of the protein. The same behaviour was also reported for the RepC protein from plasmid pT181, where substitution of residues Ser268 or Thr270 by Ala affects their DNA binding capacity, but keeps the same levels of topoisomerase activity as RepC WT (28).

These findings provide further evidence of the versatility of the pMV158 RepB protein. RepB not only displays several conformations of the OBD relative to the OD domain, but also exhibits two different DNA interaction sites within the OBD, namely the recognition helix area and the active site area.

## DATA AVAILABILITY

The atomic coordinates have been deposited in the Protein Data Bank under the accession codes: 8AMU for the OBD–DDR complex, 8AMT for the OBD stand alone structure and 8AMV for the new RepB_6_ structure.

## Supplementary Material

gkac1271_Supplemental_File

## Notes

Present address: D. Roeland Boer, ALBA synchrotron Light Source, XALOC Beamline-Experiments Division, 08290 Cerdanyola del Vallès, Barcelona, Spain.
