# Supplementary material for: Structures of pMV158 replication initiator RepB with and without DNA reveal a flexible dual-function protein
Source: Nucleic Acids Res. 2023 Jan 23;51(3):1458–72. doi: 10.1093/nar/gkac1271 (PMC9943647; doi:10.1093/nar/gkac1271)
Supplement: gkac1271_Supplemental_File [file gkac1271_supplemental_file.pdf]

SUPPLEMENTARY DATA

Table S1. List of oligonucleotides.

NAME	DNA SEQUENCE
27-mer	5’-TGCTTCCGTACTACG/ACCCCCCATTAA[Cy5]-3’
30-mer	5’-TACTGCGGAATTCTGCTTCCATCTACTACG-3’
Nick	5'-TACTACGA/CCCCCCC-3'
23AB	5’-TCGGCGACTTTTCGGCGACTTTT-3’<br>3’-AGCCGCTGAAAAGCCGCTGAAAA-5’
DDRC	5’-*CTTTTCGGCGACTTTTCGG-3’<br>3'-GAAAAGCCGCTGAAAAGCC-5'
DDRL	5’-*AATCTCGGCGACTTTTCGG-3’<br>3’-TTAGAGCCGCTGAAAAGCC-5’
DDRR	5’-*CTTTTCGGCGACTTTTTAG-3’<br>3’-GAAAAGCCGCTGAAAAATC-5’
1DDR	5’-*AATCTCGGCGACTTTTTAGA-3’<br>3'-TTAGAGCCGCTGAAAAATCT-5'
3DDR	5’-*AATCTCGGCGACTTTTCGGCGACTTTTCGGCGACTTTTTAGA-3’<br>3’-TTAGAGCCGCTGAAAAGCCGCTGAAAAGCCGCTGAAAAATCT-5’
NS	5’-*CGGGTCGGTAATTGGGTTT-3’<br>3'-GCCCAGCCATTAACCCAAA-5'

Where “/” indicates the OBD nick-site; [Cy5] indicates the fluorescent dye used to label the oligo 27-mer in the 3’ end; and “*” indicates the end and the strand of the dsDNA oligo used to label with [Cy5] (DDRC, DDRL, DDRR, 1DDR, NS and 3DDR), biotin (DDRC) or ^{32}P (3DDR).

Table S2. X-ray data collection, structure determination and refinement statistics.

Data collection	OBD-23AB	OBD alone	full-length RepB
PDB code	8AMU	8AMT	8AMV
X-ray source	XALOC (ALBA)	XALOC (ALBA)	ID30A-3 (ESRF)
Space group	$P2_1$	$P2_12_12_1$	$P2_1$
Unit cell parameters	a = 49.2; b = 33.6; c = 289.7	a = 44.5; b = 48.1; c = 73.7	a = 90.9, b = 79.3, c = 114.9
λ (Å)	0.979	0.979	0.968
Resolution range (Å)	24.7-3.0	32.66-1.50	45.52-2.77
R_{merge} (%)	0.164 (0.530)	0.186 (1.387)	0.083 (1.097)
No. of reflections:			
Total	41499 (4032)	349597 (17137)	182466 (16316)
Unique	17954 (1433)	26009 (1268)	40206 (3702)
Completeness (%)	90.5 (73.1)	99.8 (99.6)	99.3 (93.6)
$<I / \sigma(I)>$	3.7 (1.4)	9.1 (3.4)	11.8 (1.2)
Average multiplicity	2.3 (2.0)	13.4 (13.5)	4.5 (4.4)
**Refinement**			
R_{work}/R_{free}	23.7 / 28.7	15.5 / 17.5	22.1 / 26.7
No. atoms:			
Protein	4293	1155	9533
dsDNA	1874	0	0
r.m.s. deviation from target values:			
Bond lengths (Å)	0.014	0.015	0.011
Bond angle distances (Å)	1.8	1.9	1.4
B-factor from Wilson plot (Å^2)	69.6	12.5	88.1
Ramachandran plot:			
Favoured (%)	95.4	100	97.5
Allowed (%)	4.6	0.0	2.1
Disallowed (%)	0.0	0.0	0.4

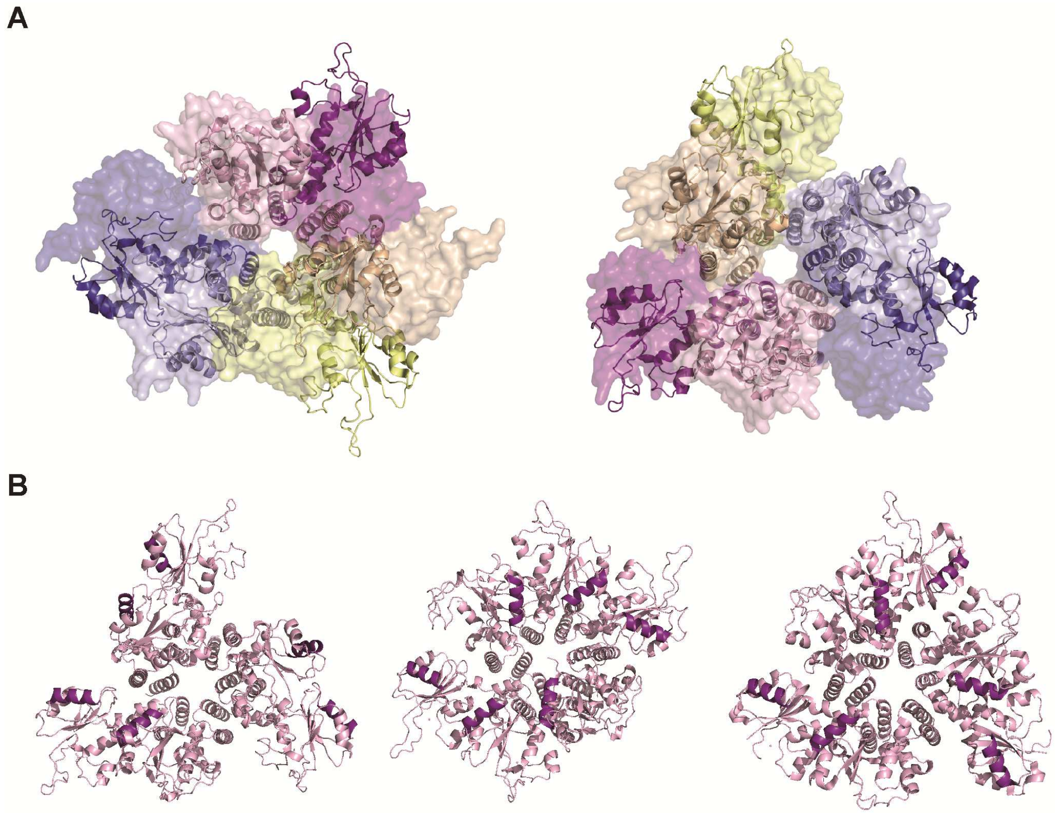

Figure S1. Comparison of the three RepB hexameric structures. (A) Superposition through the OD of the new $RepB_6$ structure described in this paper, represented by ribbons, and the 3DKX hexamer (on the left) or 3DKY hexamer (on the right), displayed by molecular surface. Each protomer is represented by a different colour. **(B)** Ribbon representation of the new $RepB_6$ structure 8AMV (left), 3DKX structure (middle) and 3DKY structure (right), in light pink, highlighting the position of α2 DNA-binding helix in deep purple.

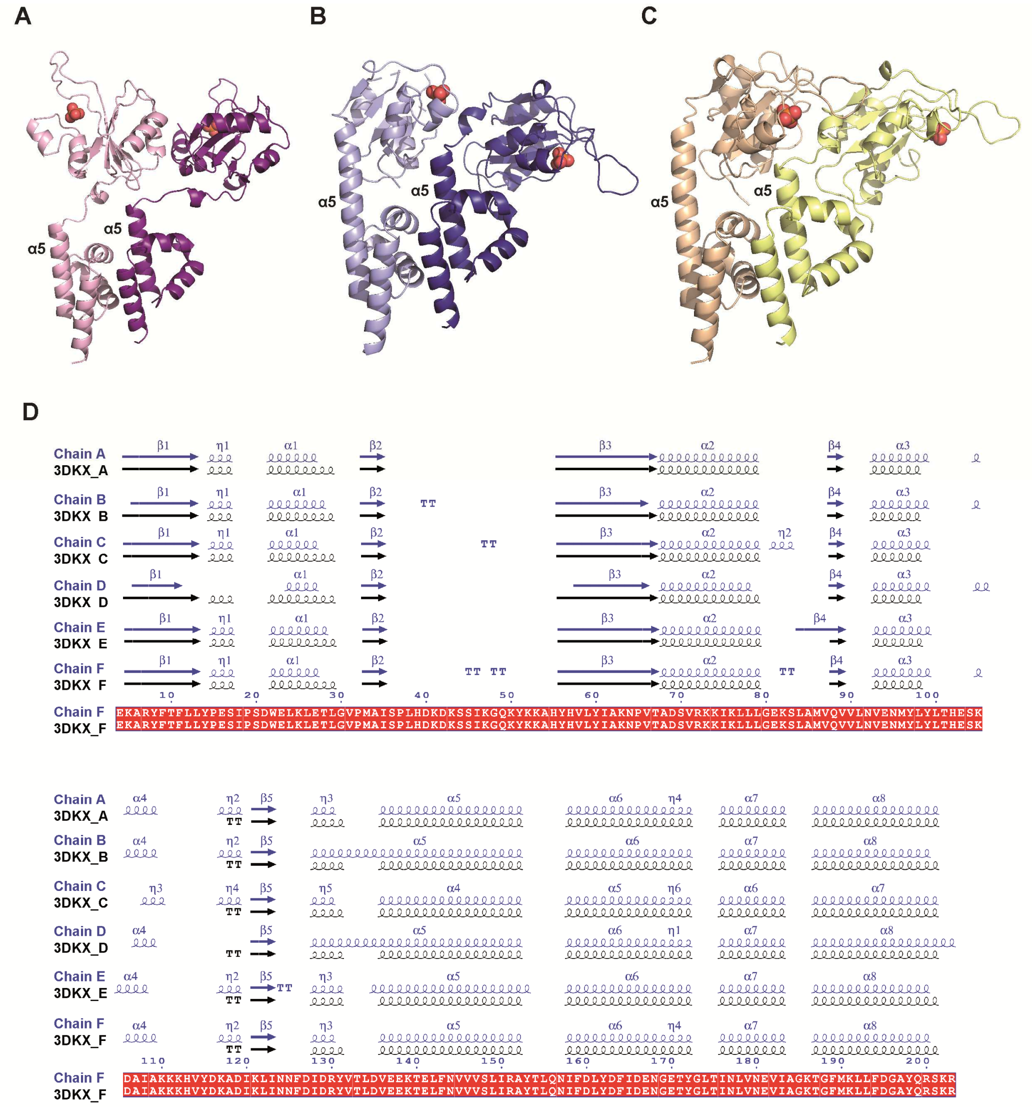

Figure S2. Cartoon representation of the RepB dimers extracted from the new hexamer. (A) Disposition of protomers A (purple) and F (pink) within the hexamer. These two protomers lack the prolonged α5 helix (indicated in the figure). **(B)** Disposition of subunit B (light blue), with the extended α5 helix (indicated in the figure) connecting both domains, and subunit C (dark blue) within the hexamer. **(C)** Disposition of protomer D (wheat), with the extended α5 helix (indicated in the figure), and protomer E (yellow) within the hexamer. In all six protomers, the phosphate molecule at the active site is represented by spheres. **(D)** Representation of the secondary structure elements of each protomer of the RepB new structure (blue) displayed above the sequence alignment against each strand in RepB 3DKX (black), performed by ENDscript (1). The symbols represent: α-helix (α and coil representation), β-strand (β and arrow), 3_{10}-helix (η and coil representation) and β-turn (TT).

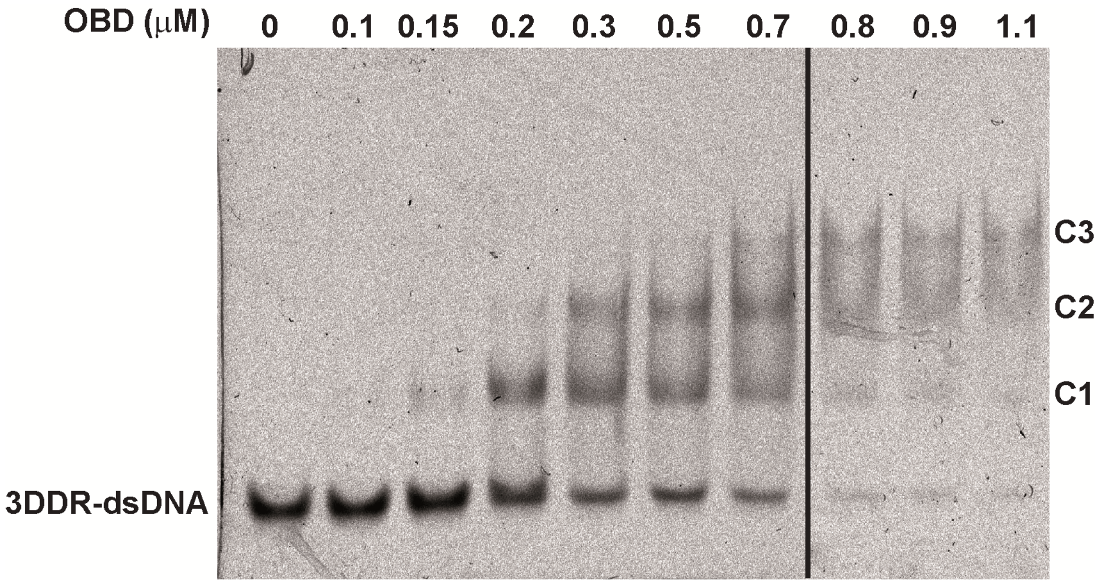

Figure S3. Analysis of the binding cooperativity of OBD to the *bind* locus. EMSA of complexes generated by binding of OBD to the 3DDR dsDNA oligo. Different concentrations of the protein, indicated on top of each gel lane, were added to fluorescently labelled 3DDR oligo (20 nM). Positions of the free DNA and of OBD–3DDR complexes C1, C2 and C3 are indicated. Images from the same gel have been grouped and indicated by a dividing line.

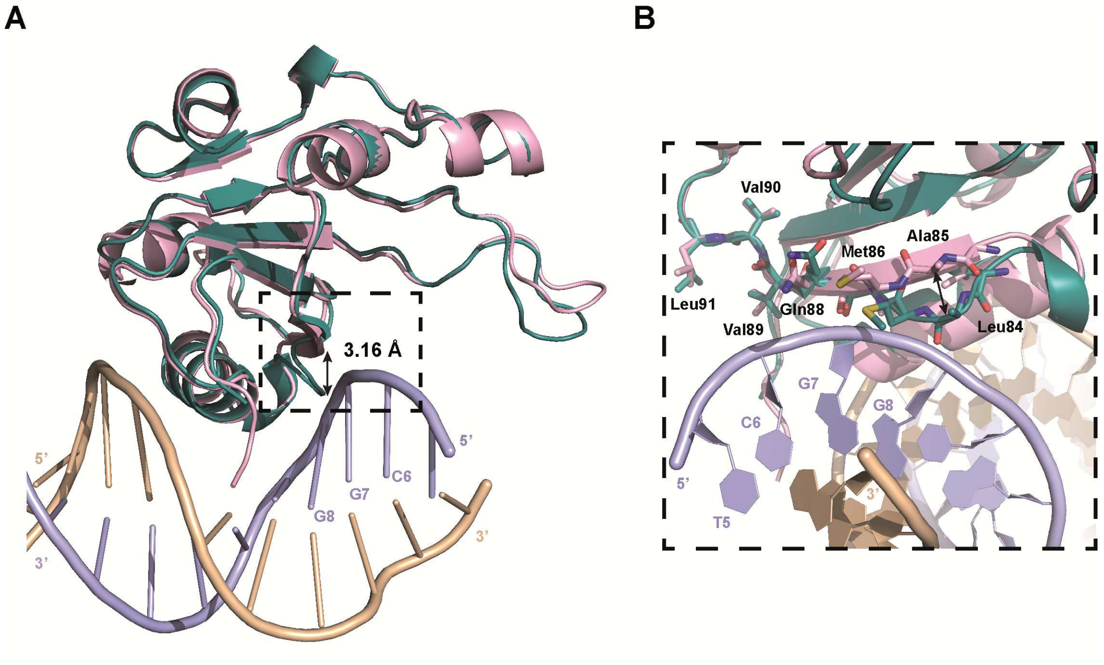

Figure S4. Superposition of the structure of the OBD-DDR complex and OBD alone. A. Ribbon representation of the superposition of the OBD-DDR complex (light pink) and OBD alone (deep teal). The loop located between helices α2 and α3 shows a displacement of 3.16 Å (indicated by an arrow) and a change to a β-strand structure (anchoring strand), upon binding of the protein to the DNA, allowing the interaction with the nucleotides highlighted in the figure,

numbered as in Figure 5A. **B.** Close-up view of the indicated region, with residues highlighted in stick representation.

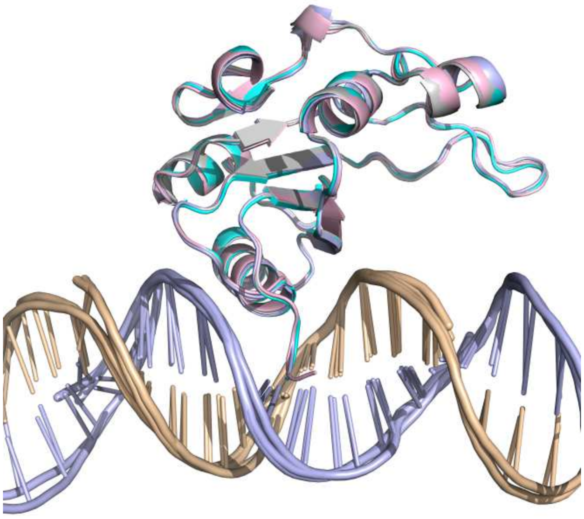

Figure S5. Binding of all OBD molecules to the DDR in the asymmetric unit. Alignment of all DDR bound OBD molecules in the asymmetric unit, displayed in ribbon with different colours. Top strand of the DNA is coloured in light blue, whilst the bottom is in wheat.

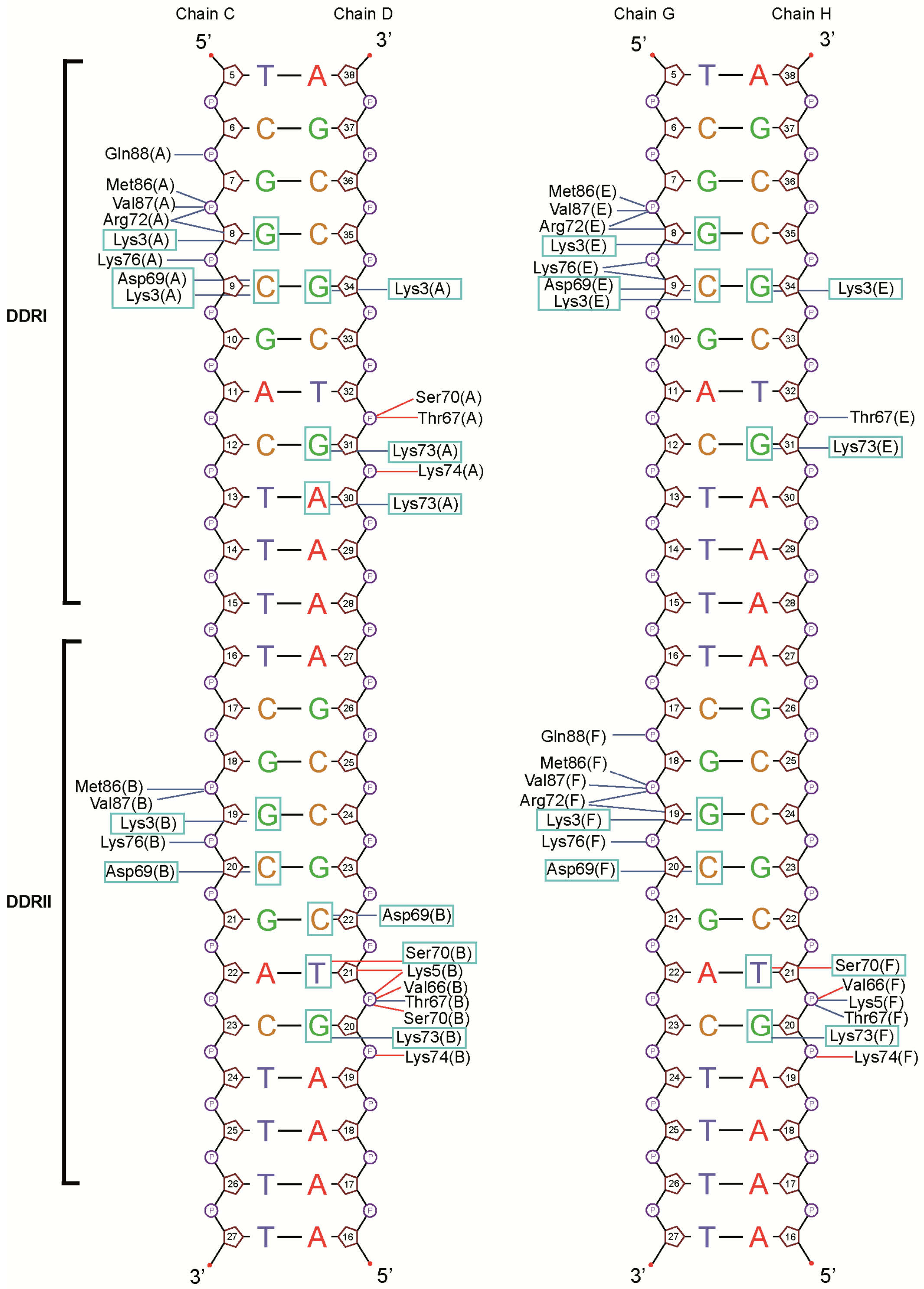

Figure S6. Protein-DNA interactions. Schematic diagram of the dsDNA structure and the protein-DNA interactions, performed with NUCPLOT (2). The DDRI and DDRII regions are delimited on the left side. Hydrogens bonds are indicated with blue lines, and van der Waals contacts with red lines. The nucleotide bases and the protein residues they interact with are both encircled with a cyan square.

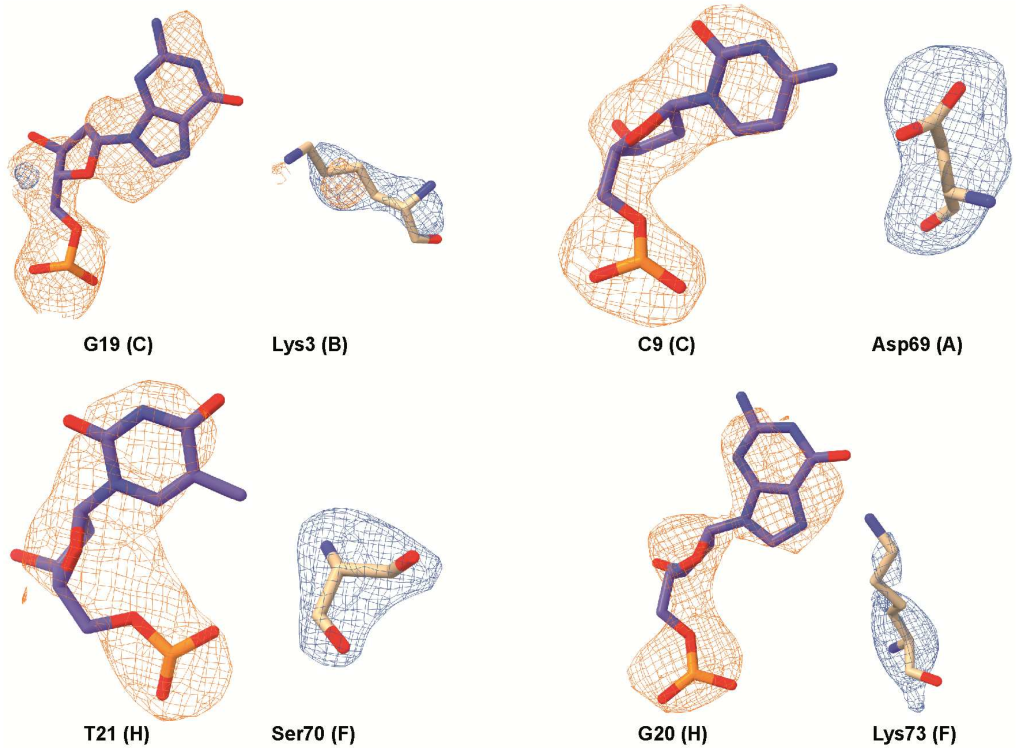

Figure S7. Omit electron density maps of bases and base interacting residues. Omit electron $|F_o|-|F_c|$ density map contoured at 2.5 σ for Lys3, Asp69, Ser70, G19, C9, T21 and G20, and contoured at 2.0 σ for Lys73. The omit map for the protein residue is coloured in blue, whilst the nucleotide one is in orange. All omit maps were calculated with Phenix (3).

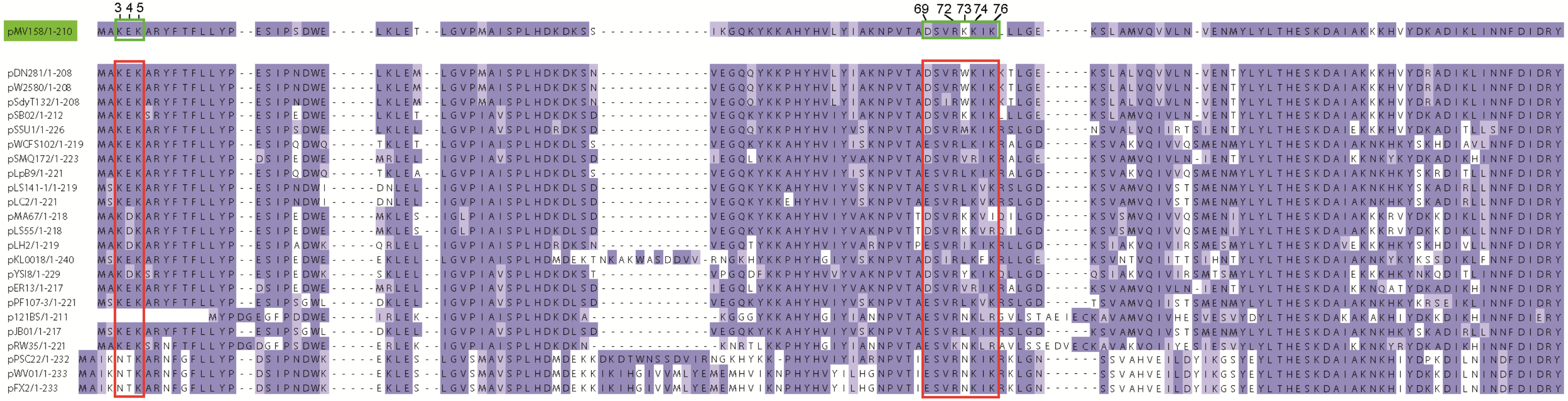

Figure S8. Comparison of OBD sequences of various plasmids of the pMV158 family. The multiple sequence alignment was performed with the Clustal Omega server in the EMBL-EBI website (https://www.ebi.ac.uk/Tools/msa/clustalo/) with default parameters. Sequences of the OBD of the replication proteins from the following plasmids are shown (accession no. in parenthesis): pMV158 (X15669), pDN281 (NC_010230), pW2580 (AY907345), pSdyT132 (NC_010907), pSB02 (AB021465), pSSU1 (NC_010907), pWCFS102 (CR377165), pSMQ172 (AF295100), pLpB9 (EU391630), pLS141-1 (AB109041.1), pLC2 (Z14234), pMA67 (DQ367664), pLS55 (NC_010375), pLH2 (X81981), pKL0018 (AB290882), pYS18 (EU185047), pER13 (NC_002776), pPF107-3 (Y12675), p121BS (NC_004957), pJB01 (AY425961), pRW35 (NC_010423), pPSC22 (X95843), pWVO1 (X56954), and pFX2 (X54310). The position number of the conserved amino acids involved in dsDNA binding is indicated on top of the pMV158-OBD sequence. The same conserved sequences are boxed in the rest of the sequences.

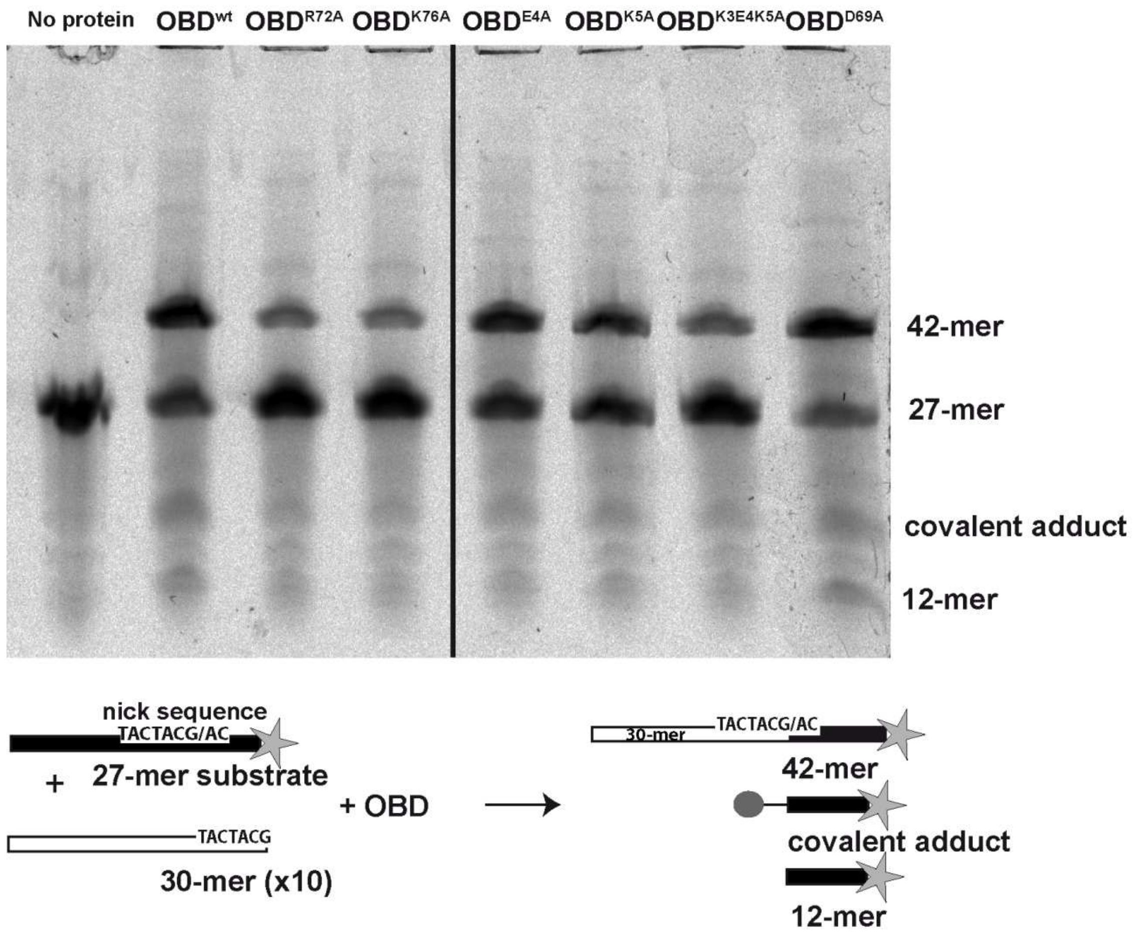

Figure S9. Nicking and strand-transfer activity of OBD^{wt} and DNA binding mutants. Reaction pattern generated by the nicking and strand-transfer activities of OBD^{WT} and OBD mutants on ssDNA oligos at a protein:oligo substrate molar ratio of 10:1. The 27-mer oligo substrate (250 nM), labeled in 3′ with the fluorescent dye Cy5 (indicated by a star), and a 10-fold molar excess of the unlabeled 30-mer oligo, were incubated with the protein at 37 °C for 1 min. The resultant fluorescent oligos were analysed by electrophoresis in 20% PAA, 8 M urea denaturing gels. A schematic description of the substrate oligos and the reaction products is depicted below the gel image, and the length of the detected oligos is also indicated. The fluorescent band corresponding to the 42-mer product resulted from the OBD-mediated strand transfer of the nicked 3'-half (12-mer) of the 27-mer substrate with the 3'-OH end provided by the 30-mer oligo. In addition, incubation of the samples with SDS and proteinase K, used to stop the reaction, allowed the detection of a covalent complex between OBD and the 12-mer oligo, which appeared as a second fluorescent band corresponding to a small peptide linked to the 5′ end of the 12-mer oligo. Hydrolisis of the covalent complex resulted in the appeareance of a third fluorescent band corresponding to a 12-mer product. Images from the same gel have been grouped and are indicated by a dividing line.

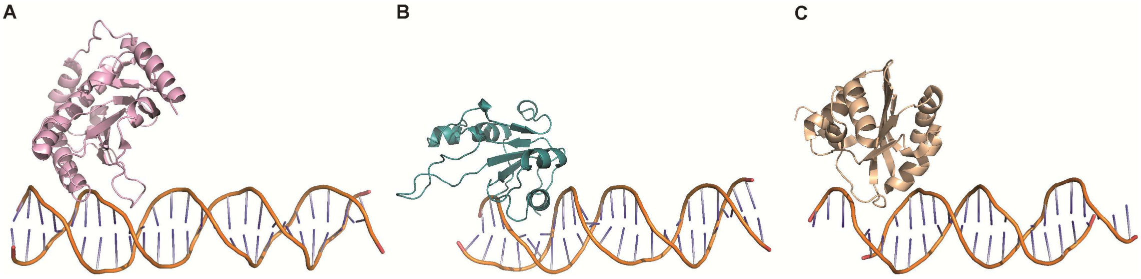

Figure S10. Comparative OBD-DNA binding strategies of the Rep proteins. Ribbon representation of the OBD of three proteins bound to dsDNA. **A.** Rep-RBS from AAV5 (light pink; 1RZ9). **B.** OBD-DDR complex, as reported here (deep teal, 8AMU). **C.** E1-DNA from papillomavirus (wheat; 1KSX).

REFERENCES

1. Robert, X. and Gouet, P. (2014) Deciphering key features in protein structures with the new ENDscript server. *Nucleic Acids Res*, **42**, W320-324.
2. Luscombe, N.M., Laskowski, R.A. and Thornton, J.M. (1997) NUCPLOT: a program to generate schematic diagrams of protein-nucleic acid interactions. *Nucleic Acids Res*, **25**, 4940-4945.
3. Liebschner, D., Afonine, P.V., Baker, M.L., Bunkoczi, G., Chen, V.B., Croll, T.I., Hintze, B., Hung, L.W., Jain, S., McCoy, A.J. *et al.* (2019) Macromolecular structure determination using X-rays, neutrons and electrons: recent developments in Phenix. *Acta Crystallogr D Struct Biol*, **75**, 861-877.
